# Comparative Efficacy and Safety of Dopamine Agonists in Advanced Parkinson's Disease With Motor Fluctuations: A Systematic Review and Network Meta-Analysis of Double-Blind Randomized Controlled Trials

**DOI:** 10.3389/fnins.2021.728083

**Published:** 2021-10-29

**Authors:** Xinglin Ruan, Fabin Lin, Dihang Wu, Lina Chen, Huidan Weng, Jiao Yu, Yingqing Wang, Ying Chen, Xiaochun Chen, Qinyong Ye, Fangang Meng, Guoen Cai

**Affiliations:** ^1^Department of Neurology, Fujian Medical University Union Hospital, Fujian, China; ^2^Department of Clinical Medicine, Fujian Medical University, Fujian, China; ^3^Beijing Neurosurgical Institute, Beijing Tiantan Hospital, Capital Medical University, Beijing, China

**Keywords:** Parkinson's disease, meta-analysis, dopamine agonist, motor fluctuations, systematic (literature) reviews

## Abstract

**Background:** Movement fluctuations are the main complication of Parkinson's disease (PD) patients receiving long-term levodopa (L-dopa) treatment. We compared and ranked the efficacy and safety of dopamine agonists (DAs) with regard to motor fluctuations by using a Bayesian network meta-analysis (NMA) to quantify information from randomized controlled trials (RCTs).

**Methods and Findings:** We carried out a systematic review and meta-analysis, and only RCTs comparing DAs for advanced PD were included. Electronic databases (PubMed, Embase, and Cochrane Library) were systematically searched for relevant studies published until January 2021. Two reviewers independently extracted individual study data and evaluated studies for risk of bias using the Cochrane Risk of Bias tool. Network meta-analyses using a Bayesian framework were used to calculate the related parameters. The pre-specified primary and secondary outcomes were efficacy (“ON” time without troublesome dyskinesia, “OFF” time, “ON” time, “UPDRS-III,” and “UPDRS-II”) and safety [treatment-emergent adverse events (TEAE) and other adverse events] of DAs. The results are presented as the surface under the cumulative ranking (SUCRA) curve. A total of 20 RCTs assessing 6,560 patients were included. The general DA effects were ranked from high to low with respect to the amount of “ON” time without troublesome dyskinesia as follows: apomorphine (SUCRA = 97.08%), pramipexole_IR (probability = 79.00%), and ropinirole_PR (SUCRA = 63.92%). The general safety of DAs was ranked from high to low with respect to TEAE as follows: placebo (SUCRA = 74.49%), pramipexole_ER (SUCRA = 63.6%), sumanirole (SUCRA = 54.07%), and rotigotine (SUCRA = 53.84%).

**Conclusions:** This network meta-analysis shows that apomorphine increased “ON” time without troublesome dyskinesia and decreased “OF” time for advanced PD patients. The addition of pramipexole, ropinirole, or rotigotine to levodopa treatment in advanced PD patients with motor fluctuations increased “ON” time without troublesome dyskinesia, improved the UPDRS III scores, and ultimately ameliorated the UPDRS II scores, thereby maximizing its benefit. This NMA of pramipexole, ropinirole, and rotigotine represents an effective treatment option and has an acceptable safety profile in patients with advanced PD.

## Introduction

Parkinson's disease (PD) is characterized by substantia nigra neurodegeneration, which causes progressive striatal dopamine deficiency and motor symptoms (Obeso et al., 2014). Parkinson's disease characterized by stages 4 and 5 of the Hoehn and Yahr scale is usually defined as advanced PD (Hoehn and Yahr, 1967). The symptoms of advanced PD include the presence of motor fluctuations, various degrees of dyskinesia, and disability with functional impact on activities of daily living and independence (Antonini et al., 2018). Motor fluctuations remain a major complication in the management of patients with PD receiving long-term levodopa (L-dopa) (Connolly and Lang, 2014; Rascol et al., 2015). Patients often report spending several hours per day in the “OFF state,” which is when they are unable to move well because of abnormal, uncontrolled, and involuntary movements, and this can substantially affect their quality of life. Therefore, controlling motor fluctuations has become a key clinical need for almost all patients with PD.

Patients with advanced PD require drugs that can maintain a strong and sustained effect or that can be added to levodopa to increase the “ON” state without troublesome dyskinesia and flatten the response for dyskinesia alleviation (Suchowersky, 2002; Aquino and Fox, 2015). Dopamine agonists (DAs) are used as an add-on therapy in addition to levodopa in advanced PD and possess excellent efficacy and acceptable side effect profiles. Thus, additional dopamine agonists (DAs) have been recommended by many guidelines as the treatment of choice for advanced Parkinson's disease patients with motor fluctuations (Grosset et al., 2010; National Institute for Health Care Excellence, 2017; Rogers et al., 2017; Fox et al., 2018; Grimes et al., 2019; Armstrong and Okun, 2020).

Despite the widespread administration of DAs as adjunctive therapy to levodopa to manage advanced PD patients with motor fluctuations, the relative comparison between the benefits and harm of the various available DAs remains unclear. Previous randomized clinical trials (RCTs) lacked direct evidence to support differences in the efficacy and safety between multiple drugs. It is a challenge, therefore, to evaluate the efficacy and safety of many or all of the available DAs for clinical indications and to select the most optimal drug. Network meta-analysis enables the comparison of different treatments by statistical inference even when some comparisons have never been evaluated in trials (Bafeta et al., 2013; Dias and Caldwell, 2019; Lin et al., 2021).

Realizing that few studies have directly compared various DAs, we performed a Bayesian network meta-analysis (NMA) of DA efficacy and attempted to assess the potential roles of apomorphine, cabergoline, pramipexole_ER, pramipexole_IR, ropinirole_IR, ropinirole_PR, rotigotine, and sumanirole. Hence, we aimed to compare and rank these eight categories of DAs for the treatment of advanced PD patients with motor fluctuations.

## Methods

### Study Design

This is a systemic review that is based on Bayesian network meta-analysis, and it has been reported according to the Preferred Reporting Items for Systematic Reviews and Meta Analyses (PRISMA) (Liberati et al., 2009; Hutton et al., 2015). Its content includes interventional measures and is based on the Cochrane handbook.

### Search Method

To perform this meta-analysis, we searched the literature for articles published up to January 2021 in three databases, including PubMed, Embase, and Cochrane library (The Cochrane Database of Systematic Reviews, CDSR). Key terms used were those relating to Parkinson's disease, motor fluctuation, therapeutic measures, and study design to identify RCTs that involved PD patients. These terms could appear anywhere in the body of the manuscript. All included studies are human trials. References accompanying the retrieved articles and relevant comment articles were also examined to identify additional related studies. In addition, we searched the WHO International Clinical Trials Registry Platform (ICTRP) for applicable studies. To reduce the language bias, the relevant literature was conducted in several languages; however, only English works were deemed suitable. The detailed search strategy appears in the Supplementary Appendix 1.

### Eligibility Criteria

1. Randomized clinical trials.
2. Studies that included advanced PD patients (PD patients classified as at stage 4 or 5 of the Hoehn and Yahr scale, which is characterized by the presence of motor fluctuations) or PD patients with motor fluctuation.
3. Studies that compared various curative measures with a placebo group or each other.
4. Studies that reported motor function, quality of life, and results of adverse events.

### Exclusion Criteria

1. Secondary studies, including general data analyses of published RCTs.
2. Studies that had <1-week follow-up duration.
3. Data that were missing or could not be extracted.
4. Studies that had insufficient data.
5. Studies that demonstrated a high risk for bias for sequence generation or allocation concealment.

### Eligibility Assessment and Data Extraction

The eligibility of each manuscript was assessed by two investigators, and disagreements were discussed with the primary researchers to reach a common conclusion. For those references, we first imported them into reference management software, deleted repetitions, and, by evaluating titles, abstracts, and full text, assessed the eligibility of the remainder of the studies. If there were several publications from the same study, we only included the most complete or most recent article.

A standardized table was designed to extract the data from eligible studies. The following variables were extracted: (1). Study information (authors, countries, publication dates, participant numbers, and study duration); (2). Patient characteristics (age, sex, basic health condition); (3). Intervention (drugs and doses); and 4. Outcomes (events/total numbers of all study participants or subgroups).

### Quality Assessment

Study quality was independently assessed by two reviewers who used the Cochrane Collaboration's risk-of-bias tool. Six fields were assessed: (i) random sequence generation, (ii) allocation concealment, (iii) blinding of participants and personnel, (iv) blinding of outcome assessment, (v) incomplete outcome data, and (vi) selective reporting. Three different evaluations were performed: (i) high risk, (ii) low risk, and (iii) unclear risk, based on the Cochrane handbook V.5.1.0, chapter 8.5 (Higgins et al., 2011). The strength of the body of evidence for the primary outcome of the meta-analyses was assessed using the Grading of Recommendations Assessment, Development, and Evaluation (GRADE) approach (Brozek et al., 2009).

### Effective Measures

The primary outcome was the mean change from baseline to endpoint in “ON” time without troublesome dyskinesia. The secondary outcome was the mean change from baseline to endpoint in “OFF” time, “ON” time, UPDRS II scores in the on-medication state, UPDRS III scores in the on-medication state, and the mean changes from baseline to endpoint in patients with at least one treatment-emergent adverse event (TEAE), including dyskinetic, fall, hallucinosis, gastrointestinal response (vomiting, diarrhea, nausea, or constipation), or other AE (headache, abnormal pain, dizziness, somnolence, or insomnia), using log odds ratio for expression.

### Statistical Analysis

The amount of the observed variance reflecting real differences in the effect size across the included trials was graded with the Q test and I^2^ statistic with values representing mild, moderate, and severe heterogeneity (<25%, 25–75%, and >75%, respectively) (Higgins et al., 2011). The variance in the true effect size across the included trials (τ^2^) was calculated. We assessed the small study effect by visual inspection of the adjusted comparison, where appropriate.

To integrate the direct and the indirect comparisons, we conducted the network meta-analysis within a Bayesian framework using Markov chain Monte Carlo (MCMC) methods in WinBUGS (MRC Biostatistics Unit, Cambridge, UK), with four chains possessing over-dispersed initial values, and Gibbs sampling based on 50,000 iterations after a burn-in phase of 20,000 iterations (Sutton and Abrams, 2001). Non-informative or vague priors for the overall mean effect [θ ~ N (0, 1002)] and the between-study standard deviation *n* [τ ~ uniform (0, 2)] were given.

We evaluated convergence according to Brooks-Gelman-Rubin. We assumed that the therapeutic effects of all included trials were the same, i.e., that the true therapeutic effects of direct and indirect analyses were the same, on average. Moreover, we also assumed that heterogeneity was normal in the network. To decide whether to use a consistency model or inconsistency model to calculate the log OR and 95% CI, we used the deviance information criterion (DIC). The DIC provides a measure of model fit that penalizes model complexity, and thus, lower values of DIC indicate a more optimal fit of the model, with a material effect for the difference value of two models (Pooley and Marion, 2018).

In addition, using the *P* value of the node split analysis, which originates from the comparison between the direct estimate value and indirect estimate value, we can evaluate the consistency of the network. *P* < 0.05 indicates significant inconsistencies that require careful interpretation. We present the relative ranking of curative effects based on drug concentration and adverse event outcomes as their surface under the cumulative ranking (SUCRA) curve, ranging from 100, indicating that the treatment has an ideal curative effect with a low incidence of adverse events, to 0, which indicates that the treatment has a poor curative effect with a relatively high incidence of adverse events. Higher SUCRA scores correspond to a higher ranking for reducing motor fluctuations and a lower risk of adverse events, compared with other interventions.

We assessed small study effects with comparison adjusted for funnel plot symmetry. We present the relative rank probability of therapeutic effects and adverse event outcomes, ranging from 1, indicating that the treatment has a high likelihood of being the most optimal, to 0, which indicates that the treatment has a high likelihood of being the least optimal. To assess the robustness of the findings of our primary efficacy outcome, we performed multiple sensitivity analyses. These included:

Exclusion of the studies that were published after 2000;

Exclusion of the studies that generated small-study effects;

Exclusion of studies with attrition bias;

Exclusion of studies with reporting bias.

Inclusion of studies that had lasted 16 weeks or more.

Also, we performed a meta-regression analysis, which included publication date of studies; sex, age, disease duration, baseline UPDRS III scores, and baseline H&Y scores of patients; and trial duration.

### Involvement of the Patients and the Public

No patients were involved in setting the research questions or the outcome measures, nor were they involved in developing plans for the design or implementation of the study. No patients were asked to advise on the study interpretation or write up the results. There are no plans to disseminate the results of the research to the study participants or the relevant patient community. It was not evaluated whether there was patient involvement with any of the studies included in the review.

## Results

### Systematic Review and Characteristics

In this meta-analysis, 20 eligible RCTs involving 6,560 patients were included (Supplementary Appendix 2). The included PD patients with motor fluctuations received eight different treatments (Guttman, 1997; Pinter et al., 1999; Mizuno et al., 2003, 2007, 2012; Wong et al., 2003; Möller et al., 2005; Barone et al., 2007; Poewe et al., 2007; Hersh et al., 2010; Schapira et al., 2011; Stocchi et al., 2011; Zhang et al., 2013, 2017; Nicholas et al., 2014; Nomoto et al., 2014; Wang et al., 2014; Zesiewicz et al., 2017; Katzenschlager et al., 2018).

**Figure 1:**
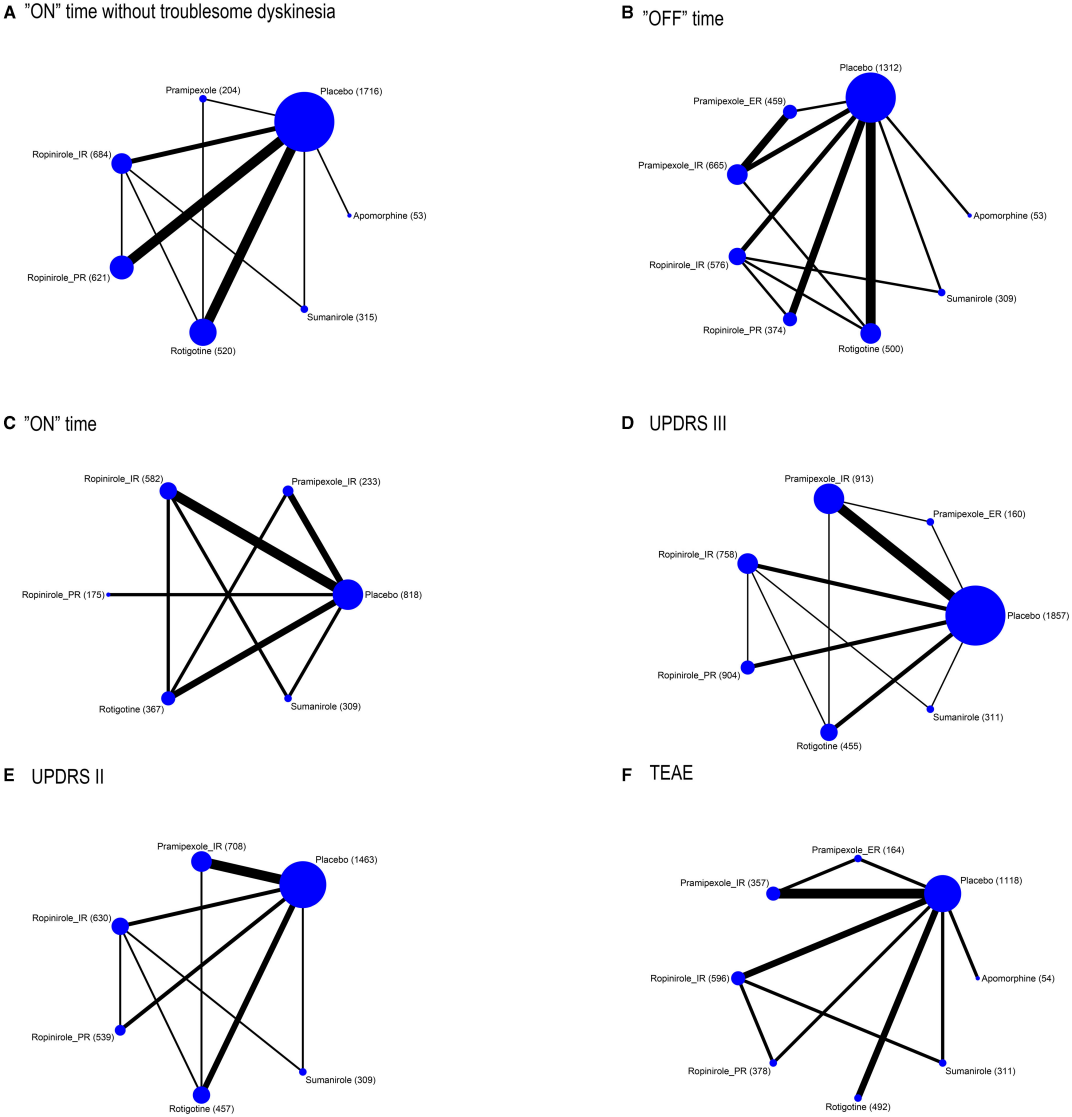
Network plot of outcomes **(A)** “on” time without troublesome dyskinesia, **(B)** “ON” time, **(C)** “OFF” time, **(D)** UPDRS III, **(E)** UPDRS II, and **(F)** TEAE. The size of the nodes corresponds to the number of participants assigned to each treatment. Treatments with direct comparisons are linked with a line; its thickness corresponds to the number of trials evaluating the comparison.

Figure 1 presents network plots of comparisons for primary and secondary outcomes. Table 1 presents the baseline characteristics of the studies. Supplementary Appendix 3 presents the risk of bias assessment for studies contributing to analyses of each outcome. Supplementary Appendix 3 also presents moderate- to high-quality evidence in primary outcomes using the GRADE approach.

**Table 1:** Baseline characteristics of patients in the studies included in the NMA.

	**References**	**Country**	**Comparison**	**Male**	**Disease duration, y, mean (SD)**	**Age, mean (SD)**	**Follow-up duration**	**Baseline H&Y, mean (SD)**	**Off time**	**Baseline UPDRS-III, mean (SD)**	**Total number**
1	Barone et al. (2007)	Italy	Placebo, *n* = 314	193	6.1^*^	65.1	40 w	2.5	NA	31.3	939
			Ropinirole, *n* = 310	194	5.6^*^	64.1		2.6		30.9	
			Sumanirole *n* = 315	187	5.7^*^	64.6		2.6		31.2	
2	Guttman (1997)	Canada	Placebo, *n* = 83	53	NA	63.72 (10.35)	24 w	NA	NA	24	246
			Pramipexole IR, *n* = 79	48		62.89 (10.03)				25	
3	Hersh et al. (2010)	US	Placebo, n = 191	NA	NA	NA	1, 2, 3, 4, 6, 8, 10, 12, 16, 20, 24w	NA	7	NA	393
			Ropinirole PR, n = 202								
4	Möller et al. (2005)	Italy	placebo, *n* = 180	122	7.9	64.7	24 w	2.43	2.43	29.8	354
			Pramipexole IR, *n* = 174	108	7.6	63.4		2.3	2.3	27.5	
5	Katzenschlager et al. (2018)	Austria	Placebo, *n* = 53	34	10.6 (4.3)	63.0 (8.3)	2, 3, 4, 6, 8, 10,12 w	NA	6.76 (2.51)	28.02 (15.25)	106
			Apomorphine, *n* = 53	32	11.8 (5.6)	63.6 (9.3)			6.69 (2.23)	30.6 (13.65)	
6	Mizuno et al. (2003)	Japan	Placebo *n* = 107	56	5.73 (7.05)	63.96 (8.64)	4 w	2.64 (0.82)	NA	27.36 (13.53)	313
			Pramipexole, *n* = 102	60	4.79 (4.07)	65.46 (9.45)		2.66 (0.70)		27.11 (12.53)	
7	Mizuno et al. (2007)	Japan	placebo, n = 120	54	5.51 (49.25)	64.7 (9.31)	16w	2.73	NA	24.9 (12.63)	241
			Ropinirole, n = 121	53	5.53 (44.86)	64.9 (9.53)		2.71		23.8 (11.04)	
8	Mizuno et al. (2012)	Japan	Pramipexole ER, n = 56	21	2.9 (2.7)	68.8 (8.0)	12w	2.41	3.1 (4.5)	24.6 (8.8)	112
			Pramipexole IR, n = 56	21	3.1 (3.5)	66.1 (7.5)		2.26	2.9 (4.3)	22.8 (9.7)	
9	Mizuno et al. (2014)	Japan	Placebo *n* = 84	42	7.0 (4.2)	65.3 (7.9)	16 w	2.8 (0.6)	4.9	25.6 (10.4)	414
			Rotigotine, *n* = 164	61	7.0 (4.9)	64.8 (8.8)		2.7 (0.6)	4.5	25.8 (10.6)	
			Ropinirole, n = 166	68	6.8 (4.2)	67.0 (7.9)		2.8 (0.6)	5	25.8 (11.0)	
10	Nicholas et al. (2014)	US	Placebo, *n* = 108	74	7.23 (3.76)	64.8 (10.2)	12 w	2.43	6.35 (2.25)	26.1 (12.5)	514
			2 mg Rotigotine, *n* = 101	77	7.51 (3.87)	65.4 (10.5)		2.42	6.37 (2.96)	25.3 (12.4)	
			4 mg Rotigotine, *n* = 107	79	7.27 (3.94)	64.6 (9.0)		2.34	6.27 (2.32)	23.1 (11.3)	
			6 mg Rotigotine, *n* = 104	73	7.79 (3.92)	64.6 (10.4)		2.42	6.39 (2.66)	24.7 (13.1)	
			8 mg Rotigotine, *n* = 94	56	7.49 (4.75)	63.2 (11.6)		2.32	6.41 (2.34)	23.9 (9.8)	
11	Nomoto et al. (2014)	Japan	Placebo, *n* = 86	44	5.4 (3.0)	66.8 (8.3)	18 w	2.7	6.0 (3.4)	26.2 (10.4)	172
			Rotigotine, *n* = 86	34	7.5 (6.0)	67.0 (6.8)		2.83	6.6 (3.5)	28.1 (12.2)	
12	Pinter et al. (1999)	Germany	Placebo, *n* = 44	31	8.5 (5.2)	60.7 (8.7)	4 w	2.95	6.8	30.5 (12.2)	78
			Pramipexole, *n* = 34	20	7.8 (4.3)	59.3 (8.3)		2.94	6.2	33.5 (9.1)	
13	Poewe et al. (2007)	Italy	Placebo, *n* = 100	71	8.5 (5.0)	NA	NA	NA	6.6 (2.8)	26.8 (11.4)	501
			Pramipexole, *n* = 200	112	8.4 (4.7)				6.0 (2.5)	26.4 (11.6)	
			Rotigotine, n = 201	132	8.9 (4.4)				6.2 (2.5)	26.3 (11.4)	
14	Schapira et al. (2011)	UK	placebo, *n* = 178	94	5.9 (3.8)	60.9 (9.7)	8,18,33 w	2.56	NA	27.7 (13.6)	517
			Pramipexole ER, *n* = 164	92	6.1 (4.0)	61.6 (9.7)		2.7		29.0 (12.9)	
			Pramipexole IR, *n* = 175	95	6.6 (4.4)	62.0 (10.3)		2.88		28.3 (13.3)	
15	Stocchi et al. (2011)	UK	Ropinirole PR, n = 174	104	7.9 (4.79)	64.9 (9.20)	1, 2, 3, 4, 5, 6, 8, 10, 12, 16, 20, 24 w	2.57	6.62 (1.95)	29.0 (12.34)	343
			Ropinirole IR, *n* = 169	91	7.5 (5.04)	65.6 (9.01)		2.63	6.65 (1.91)	28.7 (13.01)	
16	Wang et al. (2014)	China	Pramipexole ER, *n* = 236	156	5.11 (3.33)	62.9 (9.1)	18 w	2.57	NA	31.7 (12.09)	475
			Pramipexole IR, n = 239	144	4.82 (3.09)	61.8 (9.03)		2.52		44.7 (15.69)	
17	Wong et al. (2003)	China	Placebo, *n* = 77	56	4.33 (0.36)	60.94 (1.11)	14 w	NA	NA	26.58 (1.47)	147
			Pramipexole, n = 73	48	4.49 (0.40)	58.84 (1.28)				26.69 (1.33)	
18	Zesiewicz et al. (2017)	US	Placebo, *n* = 74	33	NA	63.8 (10.02)	4 w	2.83	NA	31.7 (12.3)	350
			4 mg Ropinirole PR, *n* = 25	13		66.5 (7.45)		2.74		28.9 (10.74)	
			8 mg Ropinirole PR, *n* = 76	43		65.6 (9.19)		2.76		29.4 (11.36)	
			12 mg Ropinirole PR, *n* = 75	42		65.2 (9.62)		2.91		30.0 (12.71)	
			16 mg Ropinirole PR, *n* = 75	38		63.8 (9.15)		2.78		28.9 (11.58)	
			24 mg Ropinirole PR, *n* = 25	15		66.9 (7.94)		2.82		27.2 (12.03)	
19	Zhang et al. (2013)	China	Placebo, *n* = 170	65	7.98 (48.3)	63.6 (10.5)	14, 26 w	NA	7.0 (2.90)	29.3 (12.39)	345
			Ropinirole PR, *n* = 175	59	7.68 (59.9)	64.1 (9.0)			7.0 (2.89)	28.8 (13.35)	
20	Zhang et al. (2017)	China	Placebo, *n* = 172	110	NA	62.8 (9.1)	1,15, 29, 57, 85 d	NA	6.28 (2.37)	NA	346
			Rotigotine, *n* = 174	93		61.7 (8.8)			6.26 (2.46)		

*The data are expressed as the mean (SD) unless indicated otherwise;*

* *, median; PR, prolonged release; ER, extended release; IR, immediate release; NA, not available; w, week(s); d, day(s)*.

### “ON” Time Without Troublesome Dyskinesia

**Figure 2:**
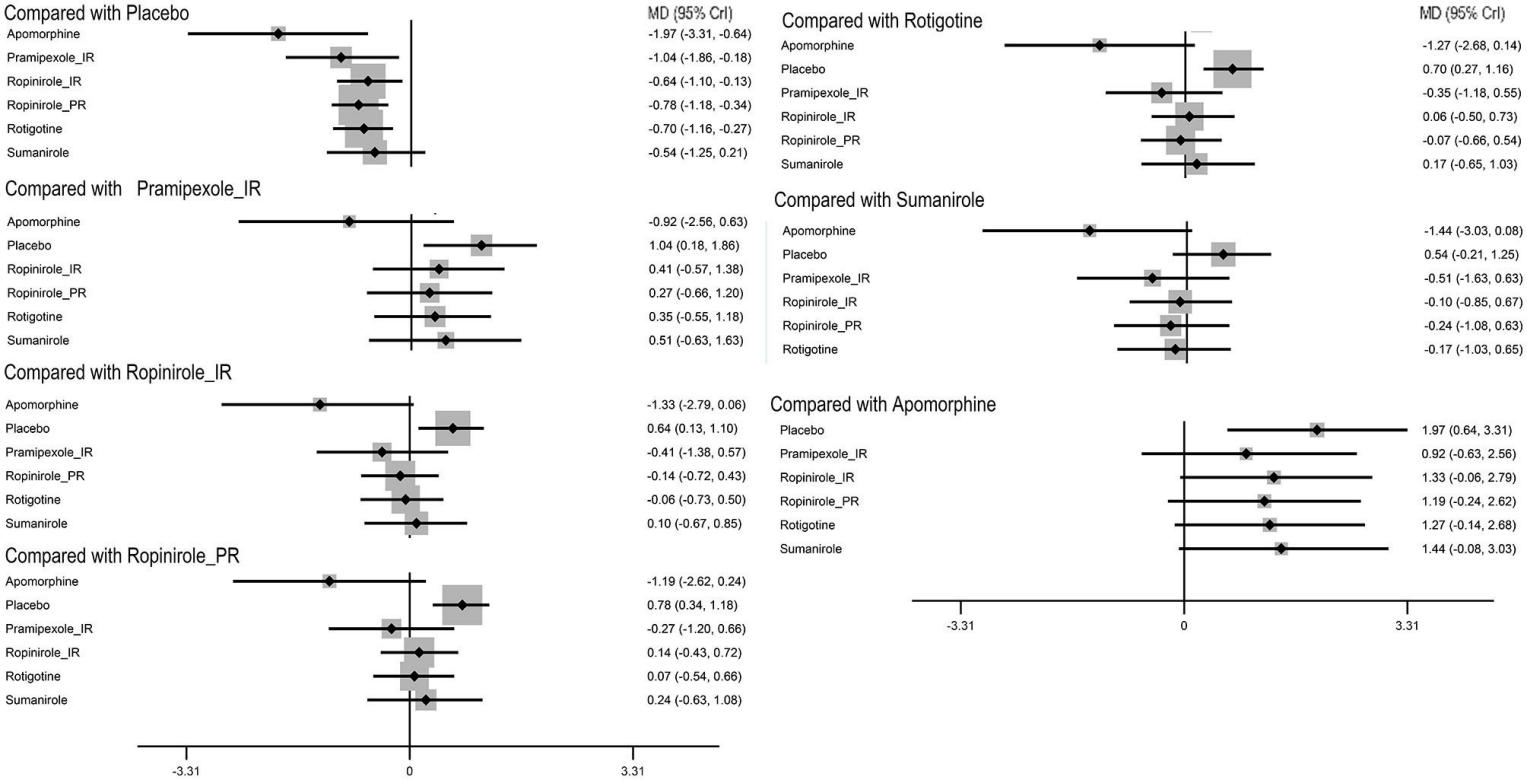
Forest plot of primary outcome ‘on’ time without troublesome dyskinesia. The size of the node corresponds to the weight in the comparison. MD, mean difference; CrI, credible interval.

**Figure 3:**
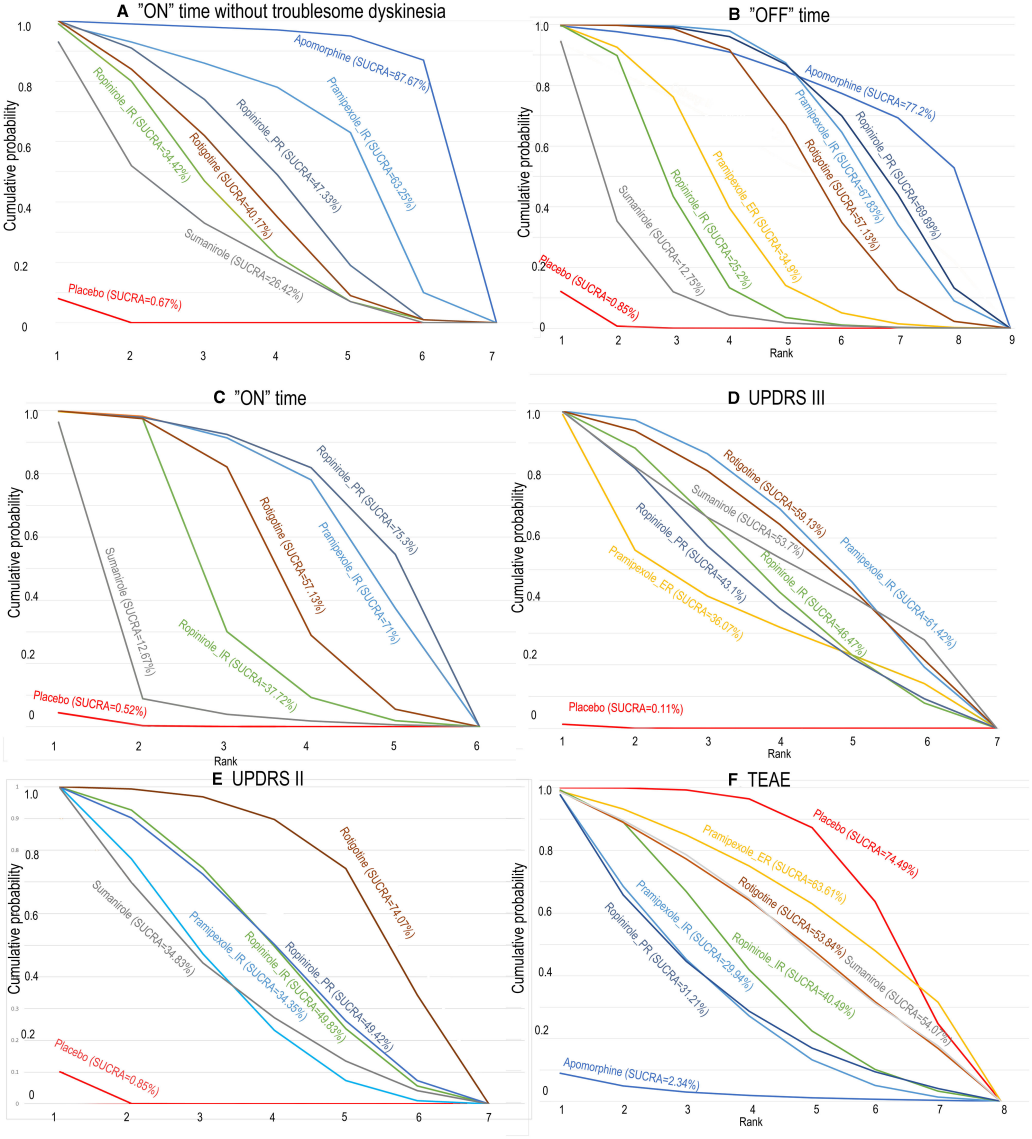
The surface under the cumulative ranking curve for competing interventions based on **(A)** “ON” time without troublesome dyskinesia, **(B)** “on” time, **(C)** “OFF” time, **(D)** UPDRS III, **(E)** UPDRS II, and **(F)** TEAE. The *x*-axis represents the ranking, and the *y*-axis represents cumulative probabilities. The greater the surface under the cumulative ranking, the greater the benefit of the intervention. SUCRA, surface under the cumulative ranking curve.

To provide patients with increased ‘ON’ time without dyskinesia, 5/6 drugs were significantly more efficacious than the placebo [apomorphine 1.97 (0.64, 3.31), pramipexole_immediate release (IR) 1.04 (0.18, 1.86), ropinirole_IR 0.64 (0.13, 1.1), ropinirole_prolonged release (PR) 0.78 (0.34, 1.18), and rotigotine 0.7 (0.27, 1.16)] (Figure 2). No significant difference was observed among these drugs. The top three ranked drugs were apomorphine (SUCRA = 97.08%), pramipexole_IR (probability = 79.00%), and ropinirole_PR (SUCRA = 63.92%) (Figure 3A).

### “OFF” Time Defined as the Return of Parkinson's Symptoms

For decreased “OFF” time, 6/8 drugs were significantly more efficacious compared with placebo [apomorphine 8 mg 1.88 (0.48, 3.26), pramipexole_extended release (ER) 0.94 (0.34, 1.56), pramipexole_immediate release (IR) 1.45 (0.95, 1.98), ropinirole_IR 0.73 (0.29, 1.26), ropinirole_prolonged release (PR) 1.51 (0.94, 2.02), rotigotine 1.3 (0.86, 1.72)] (Table 2A). The top three ranked drugs were apomorphine (SUCRA = 77.2%), ropinirole_PR (SUCRA = 69.89%), and pramipexole_IR (SUCRA = 67.83%) (Figure 3B).

**Table 2:** NMA results for the secondary outcomes.

**A**	ON									
OFF	Apomorphine								
	−0.57 (−2.7,1.62)	Cabergoline							
	***–1.88 (−3.26,−0.48)***	−1.31 (−2.97,0.33)	Placebo		***1.69 (0.81, 2.59)***	***1.13 (0.70, 1.80)***	***1.80 (0.82, 2.78)***	***1.37 (0.74, 2.09)***	0.59 (−0.15, 1.47)
	−0.94 (−2.44,0.59)	−0.36 (−2.13,1.38)	***0.94 (0.34,1.56)***	Pramipexole_ER					
	−0.42 (−1.9,1.07)	0.15 (−1.58,1.87)	***1.45 (0.95,1.98)***	***0.52 (0.01,1.01)***	Pramipexole_IR	−0.53 (−1.47,0.53)	0.11 (−1.22,1.42)	−0.31 (−1.21,0.62)	−1.08 (−2.22,0.11)
	−1.14 (−2.58,0.36)	−0.56 (−2.29,1.15)	***0.73 (0.29,1.26)***	−0.2 (−0.95,0.59)	***−0.72 (−1.38,0)***	Ropinirole_IR	0.66 (−0.57,1.69)	0.23 (−0.58,0.91)	−0.54 (−1.41,0.2)
	−0.37 (−1.87,1.11)	0.2 (−1.56,1.92)	***1.51 (0.94,2.02)***	0.57 (−0.29,1.35)	0.06 (−0.73,0.76)	***0.78 (0.04,1.4)***	Ropinirole_PR	−0.43 (−1.59,0.81)	−1.2 (−2.41,0.12)
	−0.58 (−2.03,0.88)	0 (−1.74,1.67)	***1.3 (0.86,1.72)***	0.36 (−0.38,1.06)	−0.15 (−0.8,0.46)	0.56 (−0.06,1.1)	−0.21 (−0.87,0.49)	Rotigotine	−0.77 (−1.76,0.24)
	−1.41 (−2.92,0.14)	−0.84 (−2.61,0.92)	0.47 (−0.17,1.15)	−0.47 (−1.36,0.42)	***−0.99 (−1.81,−0.15)***	−0.26 (−0.95,0.36)	***−1.04 (−1.82,−0.16)***	***−0.83 (−1.56,−0.04)***	Sumanirole
B	UPDRS II								
UPDRS III	Cabergoline	−2.04 (−5.17, 1.06)		−0.57 (−3.73, 2.58)	−0.38 (−3.56, 2.8)	−0.37 (−3.55, 2.78)	0 (−3.2, 3.16)	−0.61 (−3.85, 2.64)	
		Placebo		***1.47 (0.99, 2)***	***1.67 (0.94, 2.37)***	***1.67 (1, 2.35)***	***2.04 (1.43, 2.68)***	***1.44 (0.5, 2.38)***	
		***4.2 (0.7,7.77)***	Pramipexole_ER						
		***5.3 (3.95,6.81)***	1.1 (−2.4,4.66)	Pramipexole_IR	0.2 (−0.68, 1.01)	0.2 (−0.65, 1.04)	0.57 (−0.15, 1.25)	−0.03 (−1.11, 1)	
		***4.83 (2.99,6.63)***	0.63 (−3.38,4.55)	−0.46 (−2.85,1.71)	Ropinirole_IR	0.01 (−0.88, 0.91)	0.38 (−0.45, 1.23)	−0.22 (−1.16, 0.72)	
		***4.7 (2.88,6.63)***	0.50 (−3.46,4.51)	−0.6 (−2.97,1.7)	−0.14 (−2.46,2.35)	Ropinirole_PR	0.37 (−0.55, 1.28)	−0.23 (−1.38, 0.89)	
		***5.25 (3.41,7.17)***	1.05 (−2.89,5)	−0.05 (−2.25,2.05)	0.42 (−1.84,2.8)	0.55 (−2.09,3.13)	Rotigotine	−0.6 (−1.69, 0.47)	
		***5.09 (1.94,8.21)***	0.88 (−3.88,5.52)	−0.21 (−3.75,3.13)	0.25 (−2.87,3.4)	0.39 (−3.27,3.9)	−0.16 (−3.78,3.37)	Sumanirole	
C									
TEAE	Apomorphine								
	***2.36(0.6,4.25)***	Placebo							
	2.28 (−0.03,4.53)	−0.08 (−1.56,1.2)	Pramipexole_ER						
	1.76 (−0.38,3.74)	−0.6 (−1.74,0.22)	−0.51 (−1.98,0.78)	Pramipexole_IR					
	1.92 (−0.11,4)	−0.44 (−1.42,0.48)	−0.35 (−1.97,1.37)	0.15 (−1.06,1.65)	Ropinirole_IR				
	1.76 (−0.33,3.96)	−0.6 (−1.75,0.56)	−0.52 (−2.22,1.39)	−0.01 (−1.34,1.67)	−0.16 (−1.28,1.03)	Ropinirole_PR			
	2.11 (−0.08,4.25)	−0.24 (−1.49,0.8)	−0.15 (−1.97,1.6)	0.36 (−1.08,1.89)	0.2 (−1.35,1.62)	0.36 (−1.38,1.89)	Rotigotine		
	2.12 (−0.09,4.39)	−0.25 (−1.62,1.08)	−0.16 (−2.02,1.85)	0.35 (−1.15,2.17)	0.19 (−1.14,1.57)	0.35 (−1.33,2)	−0.01 (−1.67,1.85)	Sumanirole	

*“*ON” time (upper triangle) and “OFF” time (lower triangle). **(A)** UPDRS III (upper triangle) and UPDRS II (lower triangle); **(B)** TEAE (lower triangle); **(C)** Estimates are presented as the mean difference and 95% confidence intervals, and MDs < 0 indicate that the treatment specified in the column is more efficacious. **(A,B)**. Estimates are presented as log-risk ratios and 95% confidence intervals. Log-risk ratios < 0 indicate that the treatment specified in the column is more efficacious; **(C)** Bold underlined results indicate statistical significance*.*

### “ON” Time

For increased ‘ON’ time, 4/5 drugs were significantly more effective than the placebo [pramipexole_IR 1.69 (0.81, 2.59), ropinirole_IR 1.13 (0.70, 1.80), ropinirole_PR 1.80 (0.82, 2.78), rotigotine 1.37 (0.74, 2.09)] (Table 2A). No significant difference in “ON” time change was observed among these drugs. The top three ranked drugs were ropinirole_PR (SUCRA = 75.30%), pramipexole_IR (SUCRA = 71.00%), and rotigotine (SUCRA = 57.13%) (Figure 3C).

### Unified Parkinson's Disease Rating Scale (UPDRS)-III in the On-Medication State

Six different treatments were significantly more effective than placebo, including pramipexole_ER 4.2 (0.7, 7.77), pramipexole_IR 5.3 (3.95, 6.81), ropinirole_IR 4.83 (2.99, 6.63), ropinirole_PR 4.7(2.88, 6.63), rotigotine 5.25 (3.41, 7.17), and sumanirole 5.09 (1.94, 8.21) (Table 2B). Pramipexole_IR (SUCRA = 61.42%) ranked first, followed by rotigotine (SUCRA = 59.13%), and then sumanirole (SUCRA = 53.70%) (Figure 3D).

### UPDRS-II in the On-Medication State

It was determined that 4/5 drugs were significantly more effective than placebo including pramipexole_IR 1.47 (0.99, 2), ropinirole_IR 1.67 (0.94, 2.37), ropinirole_PR 1.67 (1, 2.35), rotigotine 2.04 (1.43, 2.68), and sumanirole 1.44 (0.5, 2.38) (Table 2B). No significant difference in UPDRS-II in the on-medication state change was observed among these drugs. In terms of cumulative rankings of UPDRS-II time, the top three were rotigotine (SUCRA = 74.07%), cabergoline (SUCRA = 57.10%), and ropinirole_PR (SUCRA = 49.42%) (Figure 3E).

### NMA of Safety

**Figure 4:**
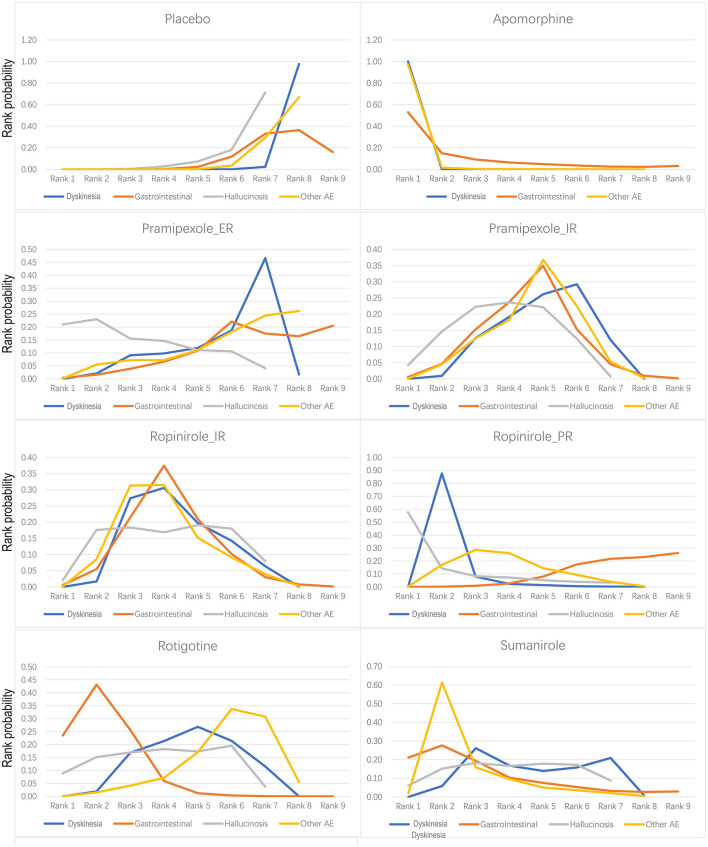
Bayesian ranking profiles of comparable treatments on safety including placebo, apomorphine, pramipexole_ER, pramipexole_IR, ropinirole_IR, ropinirole_PR, rotigotine, and sumanirole. The ranked probability score is a measure of how satisfactory forecasts that are expressed as probability distributions are in matching observed outcomes. The higher the rank, the greater the safety.

As shown in Figure 4, the treatments were ranked according to safety. Seven drugs reported results for treatment-emergent adverse events (TEAE). The log-risk ratios for apomorphine [−2.36 (−4.25, −0.6)] were significantly higher than those for the placebo (Table 2C). Pramipexole_ER, pramipexole_IR, ropinirole_IR, ropinirole_PR, rotigotine, and sumanirole did not exhibit a significantly lower or higher risk compared with placebo, with ranking results of placebo (probability = 74.49%) and pramipexole_ER (probability = 63.31%) (Figure 3F).

For dyskinetic effects, seven drugs showed a significantly higher risk compared with placebo [apomorphine −26.22 (−66.19, −7.7), pramipexole_ER −0.92 (−1.74, −0.09), pramipexole_IR −1.1 (−1.58, −0.68), ropinirole_IR −1.24 (−1.92, −0.7), ropinirole_PR −1.95 (−2.81, −1.23), rotigotine −1.15 (−1.75, −0.65), and sumanirole −1.18 (−2.11, −0.3)] (Supplementary Appendix 4A). For falls, three drugs were reported, and no evidence for significantly higher risk than placebo was observed [ropinirole_PR 0.88 (−0.37, 2.16), rotigotine 0.10 (−0.90, 1.09), and safinamide −0.63 (−1.91, 0.62)] (Supplementary Appendix 4B).

There were reported results for seven drugs with gastrointestinal response (vomiting + diarrhea + nausea + constipation). The log-risk ratios for the three drugs that were significantly higher than that of placebo were for pramipexole_IR −0.42 (−0.82, −0.05), ropinirole_IR −0.51 (−0.95,−0.02), and rotigotine −0.88 (−1.36, −0.47) (Supplementary Appendix 4C). Hallucinosis was reported for six drugs. Log-risk ratios for pramipexole_IR [−1.31 (−2.74, −0.19)] were significantly higher than placebo (Supplementary Appendix 4D). There were reported results for seven drugs that elicited other adverse events (AEs) (headache + abnormal pain + dizziness + somnolence + insomnia), with log-risk ratios for five drugs [apomorphine −2.07 (−3.69, −0.83), pramipexole_IR −0.36 (−0.65, −0.07), ropinirole_IR −0.48 (−0.81, −0.13), ropinirole_PR −0.49 (−0.87, −0.11), and sumanirole −0.68 (−1.23, −0.16)] that were significantly higher than those of placebo (Supplementary Appendix 4E).

### Consistency and Inconsistency Assessment

The consistency model fit was similar or better than that of the inconsistency model (Supplementary Appendix 5). We conducted a node-split analysis to determine inconsistencies in the primary outcome “mean difference in overall time” and the secondary outcomes “TEAE” and “Hallucinosis” (Supplementary Appendix 5).

### Small-Study Effects, Meta-Regression, and Sensitivity Analyses for the Primary Outcome

There was no evidence of small-study effects for the primary outcome and the secondary outcome (Supplementary Appendix 6). In addition, meta-regression did not reveal any significant effects of possible modifiers (Supplementary Appendix 7). However, the statistical power of these analyses was limited, and therefore, these findings should be interpreted with caution. There were no relevant deviations compared with the original NMA in the sensitivity analysis (Supplementary Appendix 8).

## Discussion

In this network meta-analysis of 20 RCTs consisting of 6,560 Parkinson's disease patients with motor fluctuations, we compared the efficacy and safety of DAs. Our analysis indicates that according to the primary outcome, the top five in the general effect category ranked from high to low were apomorphine, pramipexole_IR, ropinirole_IR, ropinirole_PR, and rotigotine. According to the TEAE result, the top five in the general safety category ranked from high to low were pramipexole_ER sumanirole, rotigotine, ropinirole_IR, ropinirole_PR, pramipexole_IR, and apomorphine.

In terms of decreasing motor symptoms (UPDRS III), we also found that pramipexole_ER, sumanirole, rotigotine, ropinirole_IR, ropinirole_PR, and pramipexole_IR were significantly effective individual therapies. For AE, the incidence of dyskinesia associated with ropinirole_PR was higher compared with the other drugs. Other AEs occurred with increased incidence for sumanirole compared with the other drugs. Rotigotine was associated with lower incidences of gastrointestinal side effects as compared to the other drugs.

### Strengths and Comparisons With Other Studies

Although traditional meta-analyses have been previously published, and network meta-analyses have compared drugs, little attention has been paid to the treatment of motor fluctuation that occurs in PD patients with a focus on comparing a limited group of classes or individual therapies (Stowe et al., 2011; Ren et al., 2014; Thorlund et al., 2014; Zhuo et al., 2017; Li et al., 2018; Mills et al., 2018; Zhao et al., 2019). In contrast to previous meta-analyses, the current analysis is the first network meta-analysis, and it integrates the broad basis of published evidence regarding randomized controlled trials to determine the efficacy and safety of drugs being used as adjuvant treatments with L-dopa for motor fluctuations in PD patients and allows a comprehensive evaluation of several categories of drugs under one overall analysis. Furthermore, this network meta-analysis also integrates evidence that is directly and indirectly compared. It has previously been reported that monoamine oxidase (MAO)-B inhibitors appear to have weaker anti-Parkinsonian effects than levodopa (Zesiewicz et al., 2017).

Our results support the findings that pramipexole, rotigotine, and ropinirole increase “ON” time without troublesome dyskinesia, improve the UPDRS III scores, and ultimately ameliorate the UPDRS II scores. However, no significant difference in efficacy was observed between these three drugs. This NMA indicates that pramipexole_IR and pramipexole_ER have similar efficacy, tolerability, and safety, which is consistent with the findings of Mizuno et al.'s study (Mizuno et al., 2012). However, we also found that pramipexole_IR ranked higher in terms of increasing the “ON” time without troublesome dyskinesia and improving motor symptoms. In addition, pramipexole_ER is convenient to take, which may improve patient compliance.

Rotigotine has a higher rank for improving activities of daily living and improves motor functions and decreases “OFF” time, which is consistent with the results of Möller et al.'s study (Möller et al., 2005). Rotigotine transdermal patches that administer continuous dopaminergic stimulation are an important treatment option for advanced PD with motor fluctuation (Nomoto et al., 2014). Rotigotine is well-tolerated and safe and does not cause any change in QTc (Malik et al., 2008). The safety profiles of the DAs were acceptable except for apomorphine. Apomorphine, ropinirole_PR, and pramipexole_IR are DA receptor agonists.

In this research, clinicians can intuitively understand the ranking of agonist drug efficacy through our research (Figure 3), clinicians can intuitively understand the safety ranking of agonist drugs through our research (Figure 4), and clinicians can provide personalized treatment plans for advanced PD patients more accurately based on rankings. For example, if a patient wants to decrease “off” time, we will choose Ropinirole_PR, instead of Ropinirole_IR. Patients with severe hallucinations should not be considered for treatment with Robinilol. We evaluated the safety of apomorphine, ropinirole_PR, and pramipexole_IR in detail, and found that ropinirole_PR and pramipexole_IR effectively reduced “OFF” time and UPDRS II, with minimal side effects. We found that the efficacy of apomorphine ranked first in terms of “ON time without troublesome dyskinesia” and “OFF time.” However, it performed poorly when considering its side effects, which were mainly TEAE consisting of gastrointestinal effects, headache, dizziness, somnolence, and insomnia.

Our analysis has several limitations. First, the number of large-scale randomized controlled trials for apomorphine regimens is relatively small. Comparisons of apomorphine regimens included in our network meta-analysis are represented by only one study, and these results should be interpreted more carefully due to the lower level of evidence. Although we are confident in our search strategy, some trials may not be included. Additionally, our study lacks long-term follow-up and trial investigation during different periods. The clinical data for several years of drug treatment are insufficient, and almost all studies in our meta-analysis did not provide information for more than 12 months.

NMA is a method that combines direct and indirect evidence for analysis, which breaks through the limitations of only two direct comparisons, and supports the complexity of comparison of multiple interventions. In fact, our network meta-analysis of head-to-head comparisons is relatively small. Consequently, the validity of the results after merging the direct and indirect evidence decreases.

It may be misleading to overemphasize the first place in probability ranking. Although one treatment ranks first, there may also be a large probability that it ranks last. Compared with other treatments, its advantages have no obvious clinical value. In addition, with a wider confidence interval, the accuracy of the ranking probability is further reduced. Therefore, a treatment may have the highest probability of being ranked first, and it is necessary to be cautious about this outcome. Assessment of change in “ON” time without troublesome dyskinesia provides a more accurate reflection of clinical response than change in “OFF” time (Hauser et al., 2000). Unfortunately, there was missing data for pramipexole_ER that could not be added to the DA comparisons. We hope that future clinical studies may provide additional data for this period.

## Conclusions

This network meta-analysis shows that apomorphine is one of the most effective agonists for motor fluctuations, and can increase “ON” time without troublesome dyskinesia and decrease “OFF” time. The addition of pramipexole, ropinirole, or rotigotine to levodopa treatment in advanced PD patients with motor fluctuations can increase “ON” time without troublesome dyskinesia, improve the UPDRS III scores, and ultimately ameliorate the UPDRS II scores, thereby maximizing its benefit. However, no significant difference in efficacy was observed between these three drugs. Pramipexole exhibited greater efficacy in terms of increasing “ON” time without troublesome dyskinesia and decreasing motor symptoms. The efficacy of rotigotine was greater in improving the quality of life. The safety of the DAs was acceptable except for apomorphine.

## Data Availability Statement

The original contributions presented in the study are included in the article/Supplementary Material, further inquiries can be directed to the corresponding authors.

## Author Contributions

All corresponding and first authors contributed to the study concept and design. All authors selected the articles and extracted the data. XR, FL, DW, and FM performed a literature search and screened articles for inclusion. XR, FL, and DW extracted data and evaluated studies for risk of bias. XR and FL analyzed, interpreted the data, and drafted the first version of the manuscript. All authors have interpreted the data, critically revised the data, provided intellectual contributions, and approved the final version of the manuscript. FL was responsible for the integrity and accuracy of the data and is the guarantor. The corresponding authors attest that all listed authors meet authorship criteria and that no others meeting the criteria have been omitted.

## Funding

This project was supported by Joint Funds for the Innovation of Science and Technology, Fujian Province (Grant No. 2018Y9011).

## Conflict of Interest

The authors declare that the research was conducted in the absence of any commercial or financial relationships that could be construed as a potential conflict of interest.

## Publisher's Note

All claims expressed in this article are solely those of the authors and do not necessarily represent those of their affiliated organizations, or those of the publisher, the editors and the reviewers. Any product that may be evaluated in this article, or claim that may be made by its manufacturer, is not guaranteed or endorsed by the publisher.
