# Supplementary material for: Comparative Efficacy and Safety of Dopamine Agonists in Advanced Parkinson's Disease With Motor Fluctuations: A Systematic Review and Network Meta-Analysis of Double-Blind Randomized Controlled Trials
Source: Front Neurosci. 2021 Oct 29;15:728083. doi: 10.3389/fnins.2021.728083 (PMC8586709; doi:10.3389/fnins.2021.728083)
Supplement: Supplementary file 1 [file Data_Sheet_1.PDF]

Appendix 1. Description of Search Strategy

Table 1. PubMed search terms

(("pramipexole "[Title/Abstract] OR "4,5,6,7-Tetrahydro-N6-propyl-2,6-benzothiazole-diamine"[Title/Abstract] OR "Pramipexol"[Title/Abstract] OR "2-Amino-4,5,6,7-tetrahydro-6-propylaminobenzothiazole"[Title/Abstract] OR "Dexpramipexole"[Title/Abstract] OR "Pramipexol, (R)-isomer"[Title/Abstract] OR "Mirapex"[Title/Abstract] OR "Pramipexol Dihydrobromide, (+-)-isomer"[Title/Abstract] OR "Pramipexol Dihydrochloride, (S)-isomer"[Title/Abstract] OR "Pramipexole Dihydrochloride Anhydrous"[Title/Abstract] OR "Sifrol"[Title/Abstract] OR "SND 919CL2x"[Title/Abstract] OR "SND919CL2x"[Title/Abstract] OR "SND-919CL2x"[Title/Abstract] OR "SND-919"[Title/Abstract] OR "SND 919"[Title/Abstract] OR "KNS 760704"[Title/Abstract] OR "KNS760704"[Title/Abstract] OR "KNS-760704"[Title/Abstract] OR "Pramipexol, (+-)-isomer"[Title/Abstract] OR "Pramipexole Dihydrochloride"[Title/Abstract] OR "Pramipexole Hydrochloride Monohydrate"[Title/Abstract] OR "2-Amino-6-propylaminotetrahydrobenzothiazole"[Title/Abstract] OR "2 Amino 6 propylaminotetrahydrobenzothiazole"[Title/Abstract] OR "6,7-Tetrahydro-N6-propyl-2,6-benzothiazolediamine dihydrochloride monohydrate") OR ("ropinirole "[Title/Abstract] OR "4-(2-(di-n-propylamino)ethyl)-2(3H)-indolone"[Title/Abstract] OR "ropinirol"[Title/Abstract] OR "SK and F 101468"[Title/Abstract] OR "SKF 101468"[Title/Abstract] OR "SK and F-101,468"[Title/Abstract] OR "Requip"[Title/Abstract] OR "ropinirole hydrochloride"[Title/Abstract]) OR ("rotigotine "[Title/Abstract] OR "2-(N-n-propyl-N-2-thienylethylamino)-5-hydroxytetralin"[Title/Abstract] OR "N 0437, hydrochloride, (S)-isomer"[Title/Abstract] OR "N 0923"[Title/Abstract] OR "N-0923"[Title/Abstract] OR "N 0924"[Title/Abstract] OR "N-0924"[Title/Abstract] OR "rotigotine, (+)-"[Title/Abstract] OR "(+)-5,6,7,8-tetrahydro-6-(propyl(2-(2-thienyl)ethyl)amino)-1-naphthol"[Title/Abstract] OR "Neupro"[Title/Abstract] OR "N 0437"[Title/Abstract] OR "N-0437"[Title/Abstract] OR "N 0437, (+-)-isomer"[Title/Abstract] OR "N 0437, (-)-isomer"[Title/Abstract] OR "N 0437, hydrochloride, (R)-isomer"[Title/Abstract] OR "rotigotine, (+--)-"[Title/Abstract] OR "racemic N-0437"[Title/Abstract] OR "rotigotine (+-)-form"[Title/Abstract] OR "1-naphthalenol, 5,6,7,8-tetrahydro-6-(propyl(2-(2-thienyl)ethyl)amino)-"[Title/Abstract] OR "(+--)-5,6,7,8-tetrahydro-6-(propyl(2-(2-thienyl)ethyl)amino)-1-naphthol"[Title/Abstract] OR "N 0437, (R)-isomer"[Title/Abstract] OR "Rotigotine CDS"[Title/Abstract]) OR ("sumanirole "[Title/Abstract] OR "U-95666E"[Title/Abstract] OR "PNU-95666"[Title/Abstract] OR "Sumanirole"[Title/Abstract] OR "5,6-dihydro-5-(methylamino)-4H-imidazo(4,5,1ij)-quinolin-2(1H)-one (Z)-2-butenedioate"[Title/Abstract]) OR ("Apomorphine"[Title/Abstract] OR "Apomorphine Hydrochloride, Anhydrous"[Title/Abstract] OR "Apomorphine Hydrochloride Anhydrous"[Title/Abstract] OR "Apomorphine Hydrochloride"[Title/Abstract] OR "Apomorphin-Teclapharm"[Title/Abstract] OR "Apomorphin Teclapharm"[Title/Abstract] OR "Apomorphine Hydrochloride, Hemihydrate"[Title/Abstract] OR "Apokinon"[Title/Abstract] OR "Apomorphine Chloride"[Title/Abstract] OR "Britaject"[Title/Abstract])) AND ((((((((((((Idiopathic Parkinson's Disease[Title/Abstract]) OR (Lewy Body Parkinson Disease[Title/Abstract])) OR (Lewy Body Parkinson's Disease[Title/Abstract])) OR (Primary Parkinsonism[Title/Abstract])) OR (Parkinsonism,Primary[Title/Abstract])) OR (Parkinson's Disease, Lewy Body[Title/Abstract])) OR (Parkinson Disease, Idiopathic[Title/Abstract])) OR (Parkinson's Disease[Title/Abstract])) OR (Parkinson's Disease, Idiopathic[Title/Abstract])) OR (Parkinson's Disease, Lewy Body[Title/Abstract])) OR (Idiopathic Parkinson Disease[Title/Abstract])) OR (Paralysis Agitans[Title/Abstract])) OR (Parkinson Disease[Title/Abstract]))

Table 2. Cochrane search terms

#1	Parkinson's disease
#2	pramipexole
#3	ropinirole
#4	rotigotine
#5	sumanirole
#6	Apomorphine
#7	#2 OR #3 OR #4 OR #5 OR #6
#8	#1 AND #7

Table 3. Embase search

#3	#1 AND #2
#2	'parkinson disease':ab,ti
#1	ropinirole:ab,ti OR pramipexole:ab,ti OR rotigotine:ab,ti OR sumanirole:ab,ti OR apomorphine:ab,ti

Appendix 2. PRISMA Diagram and Excluded Study

Figure 1. PRISMA Diagram

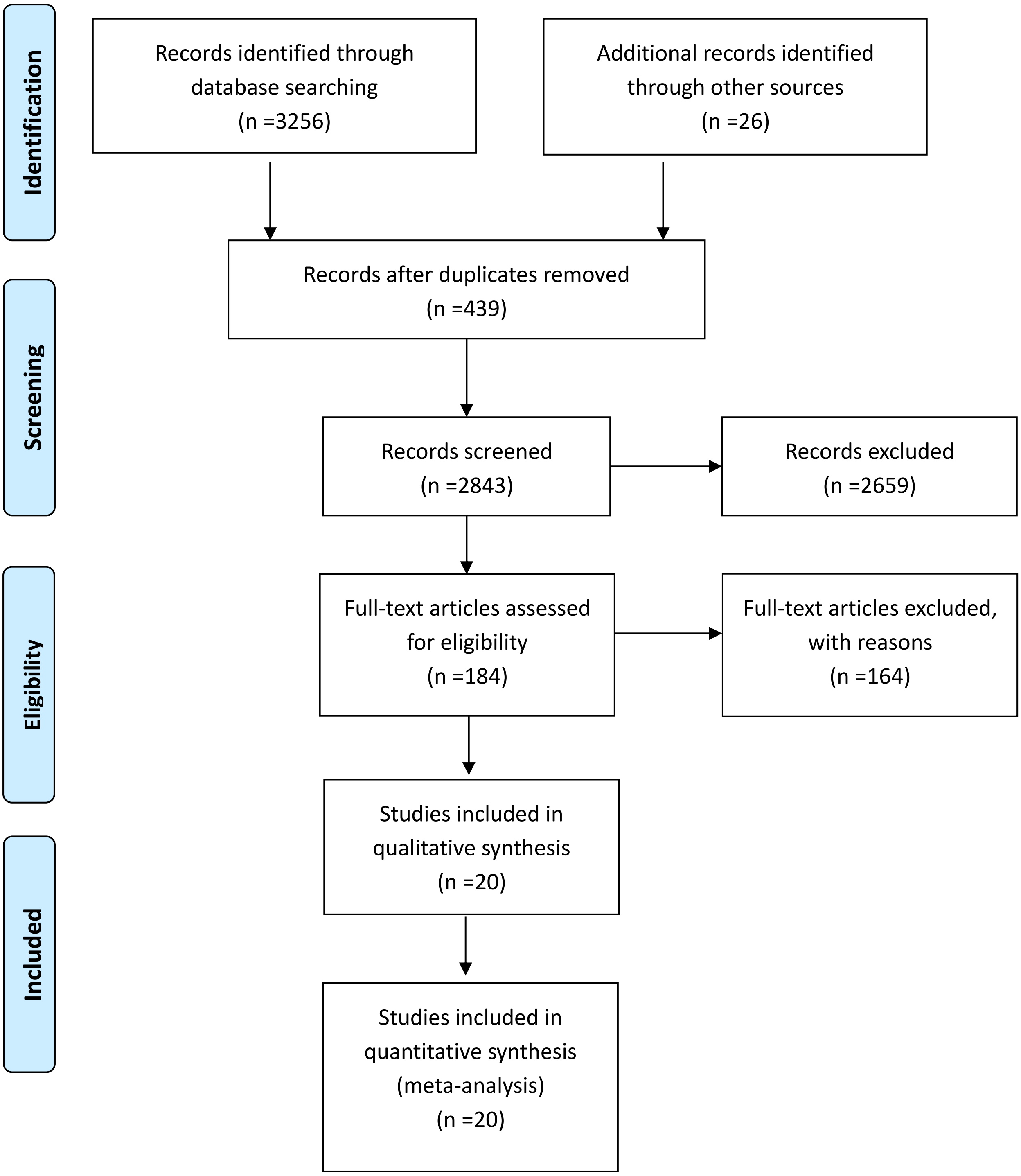

Table 1 Excluded study

No.	PMID	Title	First Author	Journal/Book	Publication Year	DOI
1	9740115	Ropinirole for the treatment of early Parkinson disease: a 12-month experience. Ropinirole Study Group	Sethi KD	Arch Neurol	1998	10.1001/archneur.55.9.1211
2	11176962	Increased growth hormone response to apomorphine in Parkinson disease compared with multiple system atrophy	Friess E	Arch Neurol	2001	10.1001/archneur.58.2.241
3	15262734	Pramipexole vs levodopa as initial treatment for Parkinson disease: a 4-year randomized controlled trial	Holloway RG	Arch Neurol	2004	10.1001/archneur.61.7.1044
4	16009751	Pathological gambling caused by drugs used to treat Parkinson disease	Dodd ML	Arch Neurol	2005	10.1001/archneur.62.9.noc50009
5	17502466	Transdermal rotigotine: double-blind, placebo-controlled trial in Parkinson disease	Jankovic J	Arch Neurol	2007	10.1001/archneur.64.5.676
6	11926889	Dopamine transporter brain imaging to assess the effects of pramipexole vs levodopa on Parkinson disease progression	Parkinson Study Group	JAMA	2002	10.1001/jama.287.13.1653
7	12838524	Slower progression of Parkinson's disease with ropinirole versus levodopa: The REAL-PET study	Whone AL	Ann Neurol	2003	10.1002/ana.10609
8	9392576	Effects of apomorphine on globus pallidus neurons in parkinsonian patients	Hutchinson WD	Ann Neurol	1997	10.1002/ana.410420513
9	21484950	Pramipexole modulates the neural network of reward anticipation	Ye Z	Hum Brain Mapp	2011	10.1002/hbm.21067
10	26626320	A Randomized Controlled Exploratory Pilot Study to Evaluate the Effect of Rotigotine Transdermal Patch on Parkinson's Disease-Associated Chronic Pain	Rascol O	J Clin Pharmacol	2016	10.1002/jcph.678
11	11391739	Transdermal dopaminergic D(2) receptor agonist therapy in Parkinson's disease with N-0923 TDS: a double-blind, placebo-controlled study	Hutton JT	Mov Disord	2001	10.1002/mds.1085
12	15390035	Continuous subcutaneous apomorphine therapy improves dyskinesias in Parkinson's disease: a prospective study using single-dose challenges	Katzenschlager R	Mov Disord	2005	10.1002/mds.20276
13	16958094	Development of dyskinesias in a 5-year trial of ropinirole and L-dopa	Rascol O	Mov Disord	2006	10.1002/mds.20988
14	16972276	Increase in body weight after pramipexole treatment in Parkinson's disease	Kumru H	Mov Disord	2006	10.1002/mds.21086
15	17094087	Valvular heart disease in Parkinson's disease patients treated with dopamine agonists: a reader-blinded monocenter echocardiography study	Junghanns S	Mov Disord	2007	10.1002/mds.21225
16	17534955	Impact of pramipexole on the onset of levodopa-related dyskinesias	Constantinescu R	Mov Disord	2007	10.1002/mds.21292
17	17318839	A comparison of sumanirole versus placebo or ropinirole for the treatment of patients with early Parkinson's disease	Singer C	Mov Disord	2007	10.1002/mds.21361
18	17935234	Rotigotine transdermal patch in early Parkinson's disease: a randomized, double-blind, controlled study versus placebo and ropinirole	Giladi N	Mov Disord	2007	10.1002/mds.21741
19	17894339	Ten-year follow-up of Parkinson's disease patients randomized to initial therapy with ropinirole or levodopa	Hauser RA	Mov Disord	2007	10.1002/mds.21743
20	19768728	An open-label conversion study of pramipexole to ropinirole prolonged release in Parkinson's disease	Lyons KE	Mov Disord	2009	10.1002/mds.22750
21	20063435	Orodispersible sublingual piribedil to abort OFF episodes: a single dose placebo-controlled, randomized, double-blind, cross-over study	Rascol O	Mov Disord	2010	10.1002/mds.22922
22	20544810	Rationale for delayed-start study of pramipexole in Parkinson's disease: the PROUD study	Schapira AH	Mov Disord	2010	10.1002/mds.23143
23	20669317	Randomized, double-blind, multicenter evaluation of pramipexole extended release once daily in early Parkinson's disease	Hauser RA	Mov Disord	2010	10.1002/mds.23317
24	20925067	Twice-daily, low-dose pramipexole in early Parkinson's disease: a randomized, placebo-controlled trial	Kieburtz K	Mov Disord	2011	10.1002/mds.23396
25	20960436	Apomorphine effect on pain threshold in Parkinson's disease: a clinical and positron emission tomography study	Dellapina E	Mov Disord	2011	10.1002/mds.23406
26	21322021	Rotigotine effects on early morning motor function and sleep in Parkinson's disease: a double-blind, randomized, placebo-controlled study (RECOVER)	Trenkwalder C	Mov Disord	2011	10.1002/mds.23441
27	23339054	Caffeine consumption and risk of dyskinesia in CALM-PD	Wills AM	Mov Disord	2013	10.1002/mds.25319
28	23801585	Transdermal rotigotine in early stage Parkinson's disease: a randomized, double-blind, placebo-controlled trial	Mizuno Y	Mov Disord	2013	10.1002/mds.25537

29	24919813	Randomized, controlled trial of rasagiline as an add-on to dopamine agonists in Parkinson's disease	Hauser RA	Mov Disord	2014	10.1002/mds.25877
30	32396257	Ropinirole Patch Versus Placebo, Ropinirole Extended-Release Tablet in Advanced Parkinson's Disease	Hattori N	Mov Disord	2020	10.1002/mds.28071
31	8474482	Apomorphine test for dopaminergic responsiveness: a dose assessment study	Bonuccelli U	Mov Disord	1993	10.1002/mds.870080207
32	9452324	Ropinirole in the treatment of early Parkinson's disease: a 6-month interim report of a 5-year levodopa-controlled study. 056 Study Group	Rascol O	Mov Disord	1998	10.1002/mds.870130111
33	9756146	A double-blind, placebo-controlled study of intranasal apomorphine spray as a rescue agent for off-states in Parkinson's disease	Dewey RB Jr	Mov Disord	1998	10.1002/mds.870130505
34	10192792	Electrophysiological and clinical desensitization to apomorphine administration in parkinsonian patients undergoing stereotaxic neurosurgery	Stefani A	Exp Neurol	1999	10.1006/exnr.1998.6990
35	10424323	Lack of pharmacokinetic interaction between ropinirole and theophylline in patients with Parkinson's disease	Thalamas C	Eur J Clin Pharmacol	1999	10.1007/s002280050632
36	15592733	A 10 year retrospective audit of long-term apomorphine use in Parkinson's disease	Tyne HL	J Neurol	2004	10.1007/s00415-004-0547-4
37	10552237	Apomorphine test: a predictor for motor responsiveness to deep brain stimulation of the subthalamic nucleus	Pinter MM	J Neurol	1999	10.1007/s004150050481
38	16607468	Pramipexole versus sertraline in the treatment of depression in Parkinson's disease: a national multicenter parallel-group randomized study	Barone P	J Neurol	2006	10.1007/s00415-006-0067-5
39	20972684	A 5-year prospective assessment of advanced Parkinson disease patients treated with subcutaneous apomorphine infusion or deep brain stimulation	Antonini A	J Neurol	2011	10.1007/s00415-010-5793-z
40	29164312	Efficacy and safety of rotigotine in elderly patients with Parkinson's disease in comparison with the non-elderly: a post hoc analysis of randomized, double-blind, placebo-controlled trials	Nomoto M	J Neurol	2018	10.1007/s00415-017-8671-0
41	30607535	Daytime sleepiness may be an independent symptom unrelated to sleep quality in Parkinson's disease	Liguori C	J Neurol	2019	10.1007/s00415-018-09179-8
42	29442177	Effectiveness of Rotigotine plus intensive and goal-based rehabilitation versus Rotigotine alone in "de-novo" Parkinsonian subjects: a randomized controlled trial with 18-month follow-up	Ferrazzoli D	J Neurol	2018	10.1007/s00415-018-8792-0
43	12658365	Pramipexole in comparison to l-dopa: a neuropsychological study	Brusa L	J Neural Transm (Vienna)	2003	10.1007/s00702-002-0811-7
44	15254792	Comparison of alpha-dihydroergocryptine and levodopa monotherapy in Parkinson's disease: assessment of changes in DAT binding with [123I]IPT SPECT	Pöpperl G	J Neural Transm (Vienna)	2004	10.1007/s00702-004-0147-6
45	10809406	Effects of apomorphine on visual functions in Parkinson's disease	Büttner T	J Neural Transm (Vienna)	2000	10.1007/s007020050007
46	9928892	Transient increase of pancreatic enzymes evoked by apomorphine in Parkinson's disease	Pinter MM	J Neural Transm (Vienna)	1998	10.1007/s007020050126
47	11145006	An open-label, multicentre clinical trial to determine the levodopa dose-sparing capacity of pramipexole in patients with idiopathic Parkinson's disease	Pinter MM	J Neural Transm (Vienna)	2000	10.1007/s007020070020
48	20535621	Transdermal rotigotine for the perioperative management of Parkinson's disease	Wüllner U	J Neural Transm (Vienna)	2010	10.1007/s00702-010-0425-4
49	21080009	Rotigotine transdermal system for control of early morning motor impairment and sleep disturbances in patients with Parkinson's disease	Giladi N	J Neural Transm (Vienna)	2010	10.1007/s00702-010-0506-4
50	23508526	The safety and tolerability of rotigotine transdermal system over a 6-year period in patients with early-stage Parkinson's disease	Giladi N	J Neural Transm (Vienna)	2013	10.1007/s00702-013-1001-5
51	25663170	Effects of L-Dopa and pramipexole on plasticity induced by QPS in human motor cortex	Enomoto H	J Neural Transm (Vienna)	2015	10.1007/s00702-015-1374-8
52	11261747	Switching from pergolide to pramipexole in patients with Parkinson's disease	Hanna PA	J Neural Transm (Vienna)	2001	10.1007/s007020170097
53	11956968	A six-month multicentre, double-blind, bromocriptine-controlled study of the safety and efficacy of ropinirole in the treatment of patients with Parkinson's disease not optimally controlled by L-dopa	Brunt ER	J Neural Transm (Vienna)	2002	10.1007/s007020200040

54	11487217	Subcutaneous continuous apomorphine infusion in fluctuating patients with Parkinson's disease: long-term results	Stocchi F	Neurol Sci	2001	10.1007/s100720170062
55	12548362	Controlled-release transdermal apomorphine treatment for motor fluctuations in Parkinson's disease	Priano L	Neurol Sci	2002	10.1007/s100720200088
56	12548370	Combination of two different dopamine agonists in the management of Parkinson's disease	Stocchi F	Neurol Sci	2002	10.1007/s100720200096
57	17657423	Use of the dopamine receptor agonist Mirapex in the treatment of Parkinson's disease	Fedorova NV	Neurosci Behav Physiol	2007	10.1007/s11055-007-0050-3
58	25773763	Pharmacokinetics, safety, and tolerability of rotigotine transdermal system in healthy Japanese and Caucasian subjects following multiple-dose administration	Cawello W	Eur J Drug Metab Pharmacokinet	2016	10.1007/s13318-015-0273-6
59	24178238	Pharmacokinetics, safety and tolerability of rotigotine transdermal patch in healthy Japanese and Caucasian subjects	Cawello W	Clin Drug Investig	2014	10.1007/s40261-013-0150-5
60	15207426	Continuous apomorphine infusion (CAI) and neuropsychiatric disorders in patients with advanced Parkinson's disease: a follow-up of two years	Morgante L	Arch Gerontol Geriatr Suppl	2004	10.1016/j.archger.2004.04.039
61	25791613	Pharmacokinetic properties and tolerability of rotigotine transdermal patch after repeated-dose application in healthy korean volunteers	Kim BH	Clin Ther	2015	10.1016/j.clinthera.2015.01.013
62	30098648	Pharmacokinetics, Tolerability, and Bioequivalence of Two Formulations of Rotigotine in Healthy Chinese Subjects	Liu Y	Clin Ther	2018	10.1016/j.clinthera.2018.05.009
63	16814808	Effects of the dopamine agonist pramipexole on depression, anhedonia and motor functioning in Parkinson's disease	Lemke MR	J Neurol Sci	2006	10.1016/j.jns.2006.05.024
64	17466338	Subcutaneous apomorphine in patients with advanced Parkinson's disease: a dose-escalation study with randomized, double-blind, placebo-controlled crossover evaluation of a single dose	Pahwa R	J Neurol Sci	2007	10.1016/j.jns.2007.03.013
65	19150100	[Continuous dopaminergic stimulation by Duodopa in advanced Parkinson's disease: Efficacy and safety]	Annic A	Rev Neurol (Paris)	2009	10.1016/j.neurol.2008.11.017
66	24915072	Non-ergot dopamine agonist rotigotine as a promising therapeutic tool in atypical parkinsonism syndromes: a 24 months pilot observational open-label study	Moretti DV	Neuropharmacology	2014	10.1016/j.neuropharm.2014.05.028
67	19428401	Dopamine and cognitive functioning in de novo subjects with Parkinson's disease: effects of pramipexole and pergolide on working memory	Costa A	Neuropsychologia	2009	10.1016/j.neuropsychologia.2009.01.039
68	17055329	Continued efficacy and safety of subcutaneous apomorphine in patients with advanced Parkinson's disease	Pfeiffer RF	Parkinsonism Relat Disord	2007	10.1016/j.parkreldis.2006.06.012
69	20605106	High compliance with rotigotine transdermal patch in the treatment of idiopathic Parkinson's disease	Schnitzler A	Parkinsonism Relat Disord	2010	10.1016/j.parkreldis.2010.06.009
70	23557594	Rotigotine and specific non-motor symptoms of Parkinson's disease: post hoc analysis of RECOVER	Ray Chaudhuri K	Parkinsonism Relat Disord	2013	10.1016/j.parkreldis.2013.02.018
71	25444083	Effects of long-term treatment with rotigotine transdermal system on dyskinesia in patients with early-stage Parkinson's disease	Giladi N	Parkinsonism Relat Disord	2014	10.1016/j.parkreldis.2014.09.016
72	27172830	Rotigotine transdermal patch in Chinese patients with early Parkinson's disease: A randomized, double-blind, placebo-controlled pivotal study	Zhang ZX	Parkinsonism Relat Disord	2016	10.1016/j.parkreldis.2016.04.022
73	28818560	Rotigotine for nocturnal hypokinesia in Parkinson's disease: Quantitative analysis of efficacy from a randomized, placebo-controlled trial using an axial inertial sensor	Bhidayasiri R	Parkinsonism Relat Disord	2017	10.1016/j.parkreldis.2017.08.010
74	26239766	Dopamine D3 receptor-preferring agonist enhances the subjective effects of cocaine in humans	Newton TF	Psychiatry Res	2015	10.1016/j.psychres.2015.07.073
75	15288710	Effect of long-term estrogen therapy on dopaminergic responsivity in post-menopausal women--a preliminary study	Craig MC	Psychoneuroendocrinology	2004	10.1016/j.psyneuen.2004.03.008
76	17239657	Efficacy and tolerability of sumanirole in restless legs syndrome: a phase II, randomized, double-blind, placebo-controlled, dose-response study	Garcia-Borreguero D	Sleep Med	2007	10.1016/j.sleep.2006.05.018

77	27448485	Rotigotine may improve sleep architecture in Parkinson's disease: a double-blind, randomized, placebo-controlled polysomnographic study	Pierantozzi M	Sleep Med	2016	10.1016/j.sleep.2016.01.016
78	11166091	Effect of apomorphine on motor and cognitive function in melancholic patients: a preliminary report	Austin MP	Psychiatry Res	2000	10.1016/s0165-1781(00)00222-5
79	9562997	Increased cortical inhibition induced by apomorphine in patients with Parkinson's disease	Manfredi L	Neurophysiol Clin	1998	10.1016/S0987-7053(97)89576-7
80	11801429	Subdyskinetic apomorphine responses in globus pallidus and subthalamus of parkinsonian patients: lack of clear evidence for the 'indirect pathway'	Stefani A	Clin Neurophysiol	2002	10.1016/s1388-2457(01)00683-6
81	20452823	Pramipexole for the treatment of depressive symptoms in patients with Parkinson's disease: a randomised, double-blind, placebo-controlled trial	Barone P	Lancet Neurol	2010	10.1016/S1474-4422(10)70106-X
82	23726851	Pramipexole in patients with early Parkinson's disease (PROUD): a randomised delayed-start trial	Schapira AH	Lancet Neurol	2013	10.1016/S1474-4422(13)70117-0
83	31818699	Apomorphine sublingual film for off episodes in Parkinson's disease: a randomised, double-blind, placebo-controlled phase 3 study	Olanow CW	Lancet Neurol	2020	10.1016/S1474-4422(19)30396-5
84	9453072	Iontophoretic delivery of apomorphine. II: An in vivo study in patients with Parkinson's disease	van der Geest R	Pharm Res	1997	10.1023/a:1012152401715
85	14653849	Differential effects of various treatment combinations on cardiovascular dysfunction in patients with Parkinson's disease	Korchounov A	Acta Neurol Scand	2004	10.1034/j.1600-0404.2003.00172.x
86	18650802	Thorough QT/QTc study in patients with advanced Parkinson's disease: cardiac safety of rotigotine	Malik M	Clin Pharmacol Ther	2008	10.1038/clpt.2008.143
87	19741594	Dopamine agonists diminish value sensitivity of the orbitofrontal cortex: a trigger for pathological gambling in Parkinson's disease?	van Eimeren T	Neuropsychopharmacology	2009	10.1038/npp.2009.124
88	23884342	Dopaminergic influences on emotional decision making in euthymic bipolar patients	Burdick KE	Neuropsychopharmacology	2014	10.1038/npp.2013.177
89	9578193	Effect of food on the pharmacokinetics of ropinirole in parkinsonian patients	Brefel C	Br J Clin Pharmacol	1998	10.1046/j.1365-2125.1998.t01-1-00704.x
90	10190658	The effect of steady-state ropinirole on plasma concentrations of digoxin in patients with Parkinson's disease	Taylor A	Br J Clin Pharmacol	1999	10.1046/j.1365-2125.1999.00867.x
91	11784342	Bilateral GPi DBS is useful to reduce abnormal involuntary movements in advanced Parkinson's disease patients, but its action is related to modality and site of stimulation	Peppe A	Eur J Neurol	2001	10.1046/j.1468-1331.2001.00302.x
92	11985633	Ropinirole for the treatment of tremor in early Parkinson's disease	Schrag A	Eur J Neurol	2002	10.1046/j.1468-1331.2002.00392.x
93	29461870	Randomized, double-blind, crossover study of the adhesiveness of two formulations of rotigotine transdermal patch in patients with Parkinson's disease	Elshoff JP	Curr Med Res Opin	2018	10.1080/03007995.2018.1430559
94	27322571	Evaluation of rotigotine transdermal patch for the treatment of depressive symptoms in patients with Parkinson's disease	Chung SJ	Expert Opin Pharmacother	2016	10.1080/14656566.2016.1202917
95	29916262	A noninterventional study evaluating the effectiveness of rotigotine and levodopa combination therapy in younger versus older patients with Parkinson's disease	Woitalla D	Expert Opin Pharmacother	2018	10.1080/14656566.2018.1480721
96	28462585	Pramipexole Modulates Interregional Connectivity Within the Sensorimotor Network	Ye Z	Brain Connect	2017	10.1089/brain.2017.0484
97	11157560	Intravenous apomorphine therapy in Parkinson's disease: clinical and pharmacokinetic observations	Manson AJ	Brain	2001	10.1093/brain/124.2.331
98	8477407	Reproducibility of motor effects induced by successive subcutaneous apomorphine injections in Parkinson's disease	Gervason CL	Clin Neuropharmacol	1993	10.1097/00002826-199304000-00003
99	8665547	Pramipexole in patients with early Parkinson's disease	Hubble JP	Clin Neuropharmacol	1995	10.1097/00002826-199508000-00006

100	8867518	Apomorphine tolerance in Parkinson's disease: lack of a dose effect	Gancher ST	Clin Neuropharmacol	1996	10.1097/00002826-199619010-00004
101	8726538	Intranasal apomorphine rescue therapy for parkinsonian "off" periods	Dewey RB Jr	Clin Neuropharmacol	1996	10.1097/00002826-199619030-00001
102	8726542	Ropinirole in the treatment of levodopa-induced motor fluctuations in patients with Parkinson's disease	Rascol O	Clin Neuropharmacol	1996	10.1097/00002826-199619030-00005
103	10047926	Efficacy and tolerability of a novel sublingual apomorphine preparation in patients with fluctuating Parkinson's disease	Ondo W	Clin Neuropharmacol	1999	10.1097/00002826-199901000-00001
104	10682229	A randomized controlled trial comparing pramipexole with levodopa in early Parkinson's disease: design and methods of the CALM-PD Study. Parkinson Study Group		Clin Neuropharmacol	2000	10.1097/00002826-200001000-00007
105	12782918	Apomorphine infusion and the long-duration response to levodopa in advanced Parkinson's disease	Stocchi F	Clin Neuropharmacol	2003	10.1097/00002826-200305000-00009
106	16855426	Rotigotine transdermal patch enables rapid titration to effective doses in advanced-stage idiopathic Parkinson disease: subanalysis of a parallel group, open-label, dose-escalation study	Babic T	Clin Neuropharmacol	2006	10.1097/01.WNF.0000228179.83335.65
107	17414939	Pramipexole in levodopa-treated Parkinson disease patients of African, Asian, and Hispanic heritage	Parkinson Study Group	Clin Neuropharmacol	2007	10.1097/01.wnf.0000240943.59617.4c
108	24992083	Influence of the nonergot dopamine agonist piribedil on vigilance in patients With Parkinson Disease and excessive daytime sleepiness (PiViCog-PD): an 11-week randomized comparison trial against pramipexole and ropinirole	Eggert K	Clin Neuropharmacol	2014	10.1097/WNF.0000000000000041
109	26536022	A Randomized Controlled Trial of Subcutaneous Apomorphine for Parkinson Disease: A Repeat Dose and Pharmacokinetic Study	Nomoto M	Clin Neuropharmacol	2015	10.1097/WNF.0000000000000111
110	18303487	Conversion from dopamine agonists to cabergoline: an open-label trial in 128 patients with advanced Parkinson disease	Linazasoro G	Clin Neuropharmacol	2008	10.1097/wnf.0b013e318067bcc4
111	18978495	Foot-tapping rate as an objective outcome measure for Parkinson disease clinical trials	Gunzler SA	Clin Neuropharmacol	2009	10.1097/WNF.0B013E3181684C22
112	18978491	Open-label study assessment of safety and adverse effects of subcutaneous apomorphine injections in treating "off" episodes in advanced Parkinson disease	LeWitt PA	Clin Neuropharmacol	2009	10.1097/WNF.0B013E31816D91F9
113	18978485	Pharmacokinetic and pharmacodynamic comparison of ropinirole 24-hour prolonged release and ropinirole immediate release in patients with Parkinson's disease	Tompson D	Clin Neuropharmacol	2009	10.1097/WNF.0B013E318176C505
114	23527823	Inhaled dry powder apomorphine (VR040) for 'off ' periods in Parkinson's disease: an in-clinic double-blind dose ranging study	Grosset KA	Acta Neurol Scand	2013	10.1111/ane.12107
115	26095948	Effects of rotigotine transdermal patch in patients with Parkinson's disease presenting with non-motor symptoms - results of a double-blind, randomized, placebo-controlled trial	Antonini A	Eur J Neurol	2015	10.1111/ene.12757
116	19220275	Antiparkinsonian drug-induced sleepiness: a double-blind placebo-controlled study of L-dopa, bromocriptine and pramipexole in healthy subjects	Micallef J	Br J Clin Pharmacol	2009	10.1111/j.1365-2125.2008.03310.x
117	15613141	Cognitive performance in people with Parkinson's disease and mild or moderate depression: effects of dopamine agonists in an add-on to L-dopa therapy	Rektorová I	Eur J Neurol	2005	10.1111/j.1468-1331.2004.00966.x
118	15613140	A randomly assigned double-blind cross-over study examining the relative anti-parkinsonian tremor effects of pramipexole and pergolide	Navan P	Eur J Neurol	2005	10.1111/j.1468-1331.2004.01019.x
119	21435111	Effect of chronic kidney disease on excessive daytime sleepiness in Parkinson disease	Baba Y	Eur J Neurol	2011	10.1111/j.1468-1331.2011.03391.x
120	22537207	Patient-reported convenience of once-daily versus three-times-daily dosing during long-term studies of pramipexole in early and advanced Parkinson's disease	Schapira AH	Eur J Neurol	2013	10.1111/j.1468-1331.2012.03712.x
121	22845710	Success rate, efficacy, and safety/tolerability of overnight switching from immediate- to extended-release pramipexole in advanced Parkinson's disease	Schapira AH	Eur J Neurol	2013	10.1111/j.1468-1331.2012.03822.x
122	16441522	Pramipexole versus levodopa in patients with early Parkinson's disease: effect on generic and disease-specific quality of life	Noyes K	Value Health	2006	10.1111/j.1524-4733.2006.00078.x

123	10478579	Nocturnal subcutaneous apomorphine infusion in Parkinson's disease and restless legs syndrome	Reuter I	Acta Neurol Scand	1999	10.1111/j.1600-0404.1999.tb00732.x
124	16629764	Effect of ropinirole on visuo-motor test in newly diagnosed Parkinson's disease patients	Badarny S	Acta Neurol Scand	2006	10.1111/j.1600-0404.2006.00601.x
125	18822087	Comparison of apomorphine and levodopa infusions in four patients with Parkinson's disease with symptom fluctuations	Nyholm D	Acta Neurol Scand	2009	10.1111/j.1600-0404.2008.01104.x
126	26861164	Teaching neurons to respond to placebos	Benedetti F	J Physiol	2016	10.1113/JP271322
127	8057129	Apomorphine induced cognitive changes in Parkinson's disease	Růzicka E	J Neurol Neurosurg Psychiatry	1994	10.1136/jnnp.57.8.998
128	9810943	Subcutaneous apomorphine in late stage Parkinson's disease: a long term follow up	Pietz K	J Neurol Neurosurg Psychiatry	1998	10.1136/jnnp.65.5.709
129	12023411	Pramipexole in patients with Parkinson's disease and marked drug resistant tremor: a randomised, double blind, placebo controlled multicentre study	Pogarell O	J Neurol Neurosurg Psychiatry	2002	10.1136/jnnp.72.6.713
130	30361296	Behavioural and trait changes in parkinsonian patients with impulse control disorder after switching from dopamine agonist to levodopa therapy: results of REIN-PD trial	Lee JY	J Neurol Neurosurg Psychiatry	2019	10.1136/jnnp-2018-318942
131	11431506	Effects of apomorphine on subthalamic nucleus and globus pallidus internus neurons in patients with Parkinson's disease	Levy R	J Neurophysiol	2001	10.1152/jn.2001.86.1.249
132	24457253	Associations between severity of motor function and nonmotor symptoms in Parkinson's disease: a post hoc analysis of the RECOVER Study	Swick TJ	Eur Neurol	2014	10.1159/000355019
133	29763930	Rotigotine Improves Abnormal Circadian Rhythm of Blood Pressure in Parkinson's Disease	Oka H	Eur Neurol	2018	10.1159/000489574
134	15939976	Anhedonia, depression, and motor functioning in Parkinson's disease during treatment with pramipexole	Lemke MR	J Neuropsychiatry Clin Neurosci	2005	10.1176/jnp.17.2.214
135	17502449	The implications of using US-specific EQ-5D preference weights for cost-effectiveness evaluation	Noyes K	Med Decis Making	2007	10.1177/0272989X07301822
136	32735501	Efficacy of pramipexole combined with levodopa for Parkinson's disease treatment and their effects on QOL and serum TNF-α levels	Huang J	J Int Med Res	2020	10.1177/0300060520922449
137	23176073	Evaluation of the safety and tolerability of rasagiline in the treatment of the early stages of Parkinson's disease	Viallet F	Curr Med Res Opin	2013	10.1185/03007995.2012.752351
138	25772231	Caregivers' and physicians' attitudes to rotigotine transdermal patch versus oral Parkinson's disease medication: an observational study	Sieb JP	Curr Med Res Opin	2015	10.1185/03007995.2015.1030376
139	21831297	Overnight switch from ropinirole to transdermal rotigotine patch in patients with Parkinson disease	Kim HJ	BMC Neurol	2011	10.1186/1471-2377-11-100
140	24004540	Comparison of once-daily versus twice-daily combination of ropinirole prolonged release in Parkinson's disease	Yun JY	BMC Neurol	2013	10.1186/1471-2377-13-113
141	24602411	Rotigotine transdermal system and evaluation of pain in patients with Parkinson's disease: a post hoc analysis of the RECOVER study	Kassubek J	BMC Neurol	2014	10.1186/1471-2377-14-42
142	25879416	Rotigotine transdermal system as add-on to oral dopamine agonist in advanced Parkinson's disease: an open-label study	Kim JM	BMC Neurol	2015	10.1186/s12883-015-0267-7
143	27267880	Evaluation of rotigotine transdermal patch for the treatment of apathy and motor symptoms in Parkinson's disease	Hauser RA	BMC Neurol	2016	10.1186/s12883-016-0610-7
144	16116131	rTMS of supplementary motor area modulates therapy-induced dyskinesias in Parkinson disease	Koch G	Neurology	2005	10.1212/01.wnl.0000172861.36430.95
145	17030757	Dopamine agonists and cardiac valvulopathy in Parkinson disease: a case-control study	Yamamoto M	Neurology	2006	10.1212/01.wnl.0000238508.68593.1d

146	17404192	Ropinirole 24-hour prolonged release: randomized, controlled study in advanced Parkinson disease	Pahwa R	Neurology	2007	10.1212/01.wnl.0000258660.74391.c1
147	17438216	Advanced Parkinson disease treated with rotigotine transdermal system: PREFER Study	LeWitt PA	Neurology	2007	10.1212/01.wnl.0000259516.61938.bb
148	17620552	Risk factors for somnolence, edema, and hallucinations in early Parkinson disease	Biglan KM	Neurology	2007	10.1212/01.wnl.0000265593.34438.00
149	8628467	Limited usefulness of electroconvulsive therapy in progressive supranuclear palsy	Barclay CL	Neurology	1996	10.1212/wnl.46.5.1284
150	9222185	Clinical evaluation of pramipexole in advanced Parkinson's disease: results of a double-blind, placebo-controlled, parallel-group study	Lieberman A	Neurology	1997	10.1212/wnl.49.1.162
151	9270567	Ropinirole for the treatment of early Parkinson's disease. The Ropinirole Study Group	Adler CH	Neurology	1997	10.1212/wnl.49.2.393
152	10078747	Pramipexole in progressive supranuclear palsy	Weiner WJ	Neurology	1999	10.1212/wnl.52.4.873
153	10668714	Effects of central dopaminergic stimulation by apomorphine on speech in Parkinson's disease	Kompoliti K	Neurology	2000	10.1212/wnl.54.2.458
154	21790503	Effect of rotigotine on sleep and quality of life in Parkinson's disease patients: post hoc analysis of RECOVER patients who were symptomatic at baseline	Ghys L	Expert Opin Pharmacother	2011	10.1517/14656566.2011.604031
155	25997442	Impact of 6-month earlier versus postponed initiation of rotigotine on long-term outcome: post hoc analysis of patients with early Parkinson's disease with mild symptom severity	Timmerman n L	Expert Opin Pharmacother	2015	10.1517/14656566.2015.1049597
156	30013321	Comparison of nocturnal symptoms in advanced Parkinson's disease patients with sleep disturbances: pramipexole sustained release versus immediate release formulations	Xiang W	Drug Des Devel Ther	2018	10.2147/DDDT.S160300
157	12964891	Cabergoline, pramipexole and ropinirole used as monotherapy in early Parkinson's disease: an evidence-based comparison	Inzelberg R	Drugs Aging	2003	10.2165/00002512-200320110-00006
158	16336019	Pramipexole and levodopa in early Parkinson's disease: dynamic changes in cost effectiveness	Noyes K	Pharmacoeconomi cs	2005	10.2165/00019053-200523120-00009
159	21963735	Pramipexole reduces the prevalence of fatigue in patients with Parkinson's disease	Morita A	Intern Med	2011	10.2169/internalmedicine.50.5456
160	23370739	Evaluation of the efficacy of pramipexole for treating levodopa-induced dyskinesia in patients with Parkinson's disease	Utsumi H	Intern Med	2013	10.2169/internalmedicine.52.8333
161	27725534	Transdermal Patch of Rotigotine Attenuates Freezing of Gait in Patients with Parkinson's Disease: An Open-Label Comparative Study of Three Non-Ergot Dopamine Receptor Agonists	Ikeda K	Intern Med	2016	10.2169/internalmedicine.55.6808
162	28120630	A randomized, fixed-dose, dose-response study of ropinirole prolonged release in advanced Parkinson's disease	Zesiewicz TA	Neurodegener Dis Manag	2017	10.2217/nmt-2016-0038
163	21244307	Long-term, open-label study of once-daily ropinirole prolonged release in early Parkinson's disease	Hauser RA	Int J Neurosci	2011	10.3109/00207454.2010.546538
164	23938309	Inhaled apomorphine in patients with 'on-off' fluctuations: a randomized, double-blind, placebo-controlled, clinic and home based, parallel-group study	Grosset KA	J Parkinsons Dis	2013	10.3233/JPD-120142

Appendix 3. Risk of Bias Assessment and GRADE Approach Estimate

Figure 1. Risk of bias summary for individual studies

	Random sequence generation (selection bias)	Allocation concealment (selection bias)	Blinding of participants and personnel (performance bias)	Blinding of outcome assessment (detection bias)	Incomplete outcome data (attrition bias)	Selective reporting (reporting bias)	Other bias
barone2007	+	+	?	+	+	+	+
guttman1997	?	?	?	?	+	+	?
hersh2010	+	+	+	+	+	+	+
J. Carsten Mo¨ller2005	+	+	+	+	+	+	+
katzenschlager2018	?	?	?	?	+	+	?
mizuno2003	+	+	+	+	+	+	+
mizuno2007 2	?	?	?	?	?	?	?
mizuno2012	+	+	+	+	+	+	?
mizuno2014	?	?	?	?	+	+	?
nicholas2014	+	+	+	+	+	+	+
nomoto2014	?	?	?	?	+	+	?
Pinter1999	?	?	?	?	+	+	?
poewe2002	+	?	+	?	+	+	?
poewe2007	+	+	+	+	+	+	+
schapira2011	+	+	+	+	+	+	+
stocchi2011	?	+	?	+	+	+	?
wang2014	+	+	+	+	+	+	+
wong2003	?	?	?	?	+	+	?
Zesiewicz2017	+	+	?	+	+	+	?
Zhang2013	+	+	+	?	+	+	+
zhang2017	+	+	+	+	+	+	+

Figure 2 Risk of bias graph: review authors' judgements about each risk of bias item presented as percentages across all included studies.

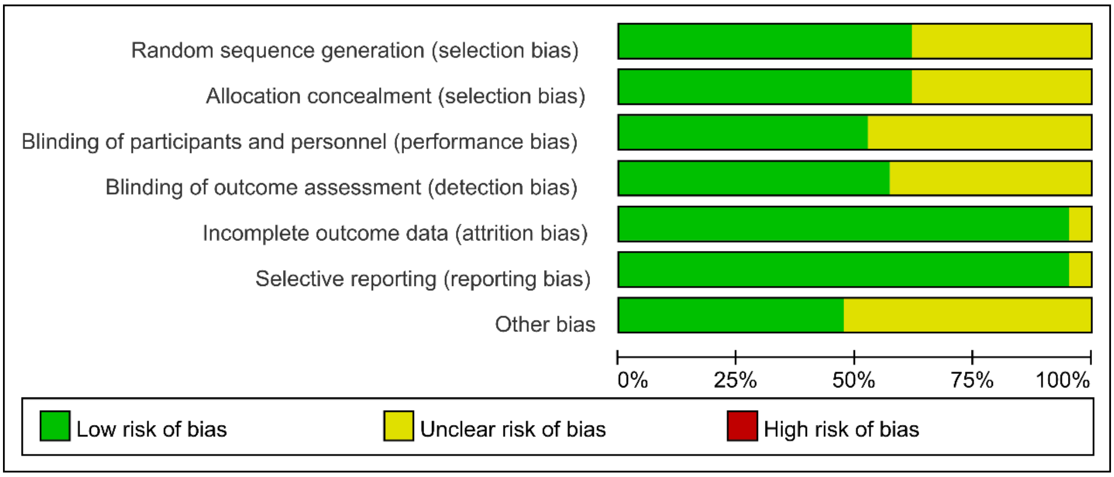

Table 1. Reasons for review authors' judgements

Characteristics of studies

Characteristics of included studies

barone2007

Methods	
Participants	
Interventions	
Outcomes	
Notes	

Risk of bias table

Bias	Authors' judgement	Support for judgement
Random sequence generation (selection bias)	Low risk	Eligible subjects were assigned treatment according to a randomization schedule (using a 1:1:1 allocation scheme and stratification with respect to selegiline use) controlled by an Interactive Voice Response System (IVRS).
Allocation concealment (selection bias)	Low risk	Unlikely to happen.
Blinding of participants and personnel (performance bias)	Unclear risk	Lack of sufficient information to determine the risk of bias
Blinding of outcome assessment (detection bias)	Low risk	Outcomes are unlikely to be affected by blindness
Incomplete outcome data (attrition bias)	Low risk	Unlikely to happen.
Selective reporting (reporting bias)	Low risk	There is no obvious other bias.
Other bias	Low risk	There is no obvious other bias.

guttman1997

Methods	
Participants	
Interventions	
Outcomes	
Notes	

Risk of bias table

Bias	Authors' judgement	Support for judgement
Random sequence generation (selection bias)	Unclear risk	not mentioned in the paper
Allocation concealment (selection bias)	Unclear risk	not mentioned in the paper
Blinding of participants and personnel (performance bias)	Unclear risk	not mentioned in the paper
Blinding of outcome assessment (detection bias)	Unclear risk	not mentioned in the paper
Incomplete outcome data (attrition bias)	Low risk	patients had assessments
Selective reporting (reporting bias)	Low risk	all results were covered
Other bias	Unclear risk	unlikely to happen

mizuno2003

Methods	
Participants	
Interventions	
Outcomes	
Notes	

Risk of bias table

Bias	Authors' judgement	Support for judgement
Random sequence generation (selection bias)	Low risk	Using computer generated code prepared by the external statistician.
Allocation concealment (selection bias)	Low risk	Using identical placebo tablets.
Blinding of participants and personnel (performance bias)	Low risk	All study personnel and participants were blinded to the study medication.
Blinding of outcome assessment (detection bias)	Low risk	Outcomes are unlikely to be affected by blindness
Incomplete outcome data (attrition bias)	Low risk	No missing data for the outcome
Selective reporting (reporting bias)	Low risk	Missing outcome data balanced in numbers across intervention groups, with similar reasons for missing data across groups
Other bias	Low risk	There is no obvious other bias.

Selective reporting (reporting bias)	Low risk	The study protocol is available and all of the study ’ s pre-specified (primary and secondary) outcomes that are of interest in the review have been reported in the pre-specified way
Other bias	Unclear risk	Lack of sufficient information to determine the risk of bias

nicholas2014

Methods	
Participants	
Interventions	
Outcomes	
Notes	

Risk of bias table

Bias	Authors' judgement	Support for judgement
Random sequence generation (selection bias)	Low risk	Study investigators telephoned an IVRS to allocate patients,based on a randomization schedule produced by UCB Pharma.
Allocation concealment (selection bias)	Low risk	.Rotigotine and placebo were administered as once-daily patchesoftwodifferentsizes(10cm2 and20cm2)that wereidenticalinappearance
Blinding of participants and personnel (performance bias)	Low risk	Blinding of investigators and patients was maintained by applying upwards from one to three patches daily to achieve the assigned daily dose.
Blinding of outcome assessment (detection bias)	Low risk	Outcomes are unlikely to be affected by blindness.
Incomplete outcome data (attrition bias)	Low risk	No missing data
Selective reporting (reporting bias)	Low risk	The study protocol is available and all of the study ’ s pre-specified (primary and secondary) outcomes that are of interest in the review have been reported in the pre-specified way
Other bias	Low risk	There is no obvious other bias.

nomoto2014

Methods	
Participants	
Interventions	
Outcomes	
Notes	

Risk of bias table

Bias	Authors' judgement	Support for judgement
Random sequence generation (selection bias)	Unclear risk	Insufficient information about the sequence generation process to permit judgement of ‘‘low risk’’ or ‘‘high risk’’.
Allocation concealment (selection bias)	Unclear risk	Insufficient information to permit judgement of ‘Low risk’ or "High risk".
Blinding of participants and personnel (performance bias)	Unclear risk	Insufficient information to permit judgement of ‘Low risk’ or "High risk".
Blinding of outcome assessment (detection bias)	Unclear risk	Insufficient information to permit judgement of ‘Low risk’ or "High risk".
Incomplete outcome data (attrition bias)	Low risk	No missing data
Selective reporting (reporting bias)	Low risk	The study protocol is available and all of the study’s pre-specified (primary and secondary) outcomes that are of interest in the review have been reported in the pre-specified way
Other bias	Unclear risk	Lack of sufficient information to determine the risk of bias

Pinter1999

Methods	
Participants	
Interventions	
Outcomes	
Notes	

Risk of bias table

Bias	Authors' judgement	Support for judgement
Random sequence generation (selection bias)	Unclear risk	Insufficient information about the sequence generation process to permit judgement of ‘‘low risk’’ or ‘‘high risk’’.

Allocation concealment (selection bias)	Unclear risk	Insufficient reporting of attrition/exclusions to permit judgement of ‘Low risk’ or ‘High risk’
Blinding of participants and personnel (performance bias)	Unclear risk	Insufficient reporting of attrition/exclusions to permit judgement of ‘Low risk’ or ‘High risk’
Blinding of outcome assessment (detection bias)	Unclear risk	Insufficient reporting of attrition/exclusions to permit judgement of ‘Low risk’ or ‘High risk’
Incomplete outcome data (attrition bias)	Low risk	No missing data.
Selective reporting (reporting bias)	Low risk	The study protocol is available and all of the study’s pre-specified (primary and secondary) outcomes that are of interest in the review have been reported in the pre-specified way
Other bias	Unclear risk	Lack of sufficient information to determine the risk of bias

poewe2002

Methods	
Participants	
Interventions	
Outcomes	
Notes	

Risk of bias table

Bias	Authors' judgement	Support for judgement
Random sequence generation (selection bias)	Low risk	The computer-generated randomization procedure was performed separately for each centre.
Allocation concealment (selection bias)	Unclear risk	Not mentioned in the paper
Blinding of participants and personnel (performance bias)	Low risk	Only the sponsor-employed person who generated the plan was aware of a given individual’s assignment during the study.
Blinding of outcome assessment (detection bias)	Unclear risk	Not mentioned in the paper
Incomplete outcome data (attrition bias)	Low risk	All patients were covered
Selective reporting (reporting bias)	Low risk	All results mentioned in the method were covered
Other bias	Unclear risk	due to the lack of blinding evidence, other bias might occur

poewe2007

Methods	
Participants	
Interventions	
Outcomes	
Notes	

Risk of bias table

Bias	Authors' judgement	Support for judgement
Random sequence generation (selection bias)	Low risk	Randomisation was implemented by an interactive voice response system with a computerised randomisation schedule stratifi ed by centre in blocks of fi ve.
Allocation concealment (selection bias)	Low risk	Investigators were blinded to all patient treatment details, which were allocated and maintained by the interactive voice response system. Study participants were unaware of the allocated treatment.
Blinding of participants and personnel (performance bias)	Low risk	both investagtors and patients were blinded to the trial
Blinding of outcome assessment (detection bias)	Low risk	Unlikely to happen
Incomplete outcome data (attrition bias)	Low risk	All randomised patients who received at least one dose of study medication were included in the safety analysis.
Selective reporting (reporting bias)	Low risk	All results mentioned in the method were covered
Other bias	Low risk	Unlikely to happen

schapira2011

Methods	
Participants	
Interventions	
Outcomes	
Notes	

Risk of bias table

Bias	Authors' judgement	Support for judgement
Random sequence generation (selection bias)	Low risk	Treatment allocation was determined by randomization code provided by the study sponsor, using the commercial program PMX CTM (release 3.3.0; Propack Data GmbH; Karlsruhe, Germany).
Allocation concealment (selection bias)	Low risk	Using identical placebo tablets.
Blinding of participants and personnel (performance bias)	Low risk	Access to the randomization schedule was restricted to the sponsor ' s Clinical Trial Support and Clinical Trial Supplies Unit, with no access by any persons directly involved in the study ' s conduct or data analysis.
Blinding of outcome assessment (detection bias)	Low risk	Outcomes are unlikely to be affected by blindness
Incomplete outcome data (attrition bias)	Low risk	No missing data for the outcome
Selective reporting (reporting bias)	Low risk	The study protocol is available and all of the study ' s pre-specified (primary and secondary) outcomes that are of interest in the review have been reported in the pre-specified way
Other bias	Low risk	There is no obvious other bias.

stocchi2011

Methods	
Participants	
Interventions	
Outcomes	
Notes	

Risk of bias table

Bias	Authors' judgement	Support for judgement
Random sequence generation (selection bias)	Unclear risk	Insufficient reporting of attrition/exclusions to permit judgement of ' Low risk ' or ' High risk '
Allocation concealment (selection bias)	Low risk	using an interactive voice recognition system.
Blinding of participants and personnel (performance bias)	Unclear risk	Insufficient information to permit judgement of ' Low risk ' or ' High risk ' .
Blinding of outcome assessment (detection bias)	Low risk	Outcomes are unlikely to be affected by blindness

Incomplete outcome data (attrition bias)	Low risk	No missing data for the outcome
Selective reporting (reporting bias)	Low risk	The study protocol is available and all of the study ˀ s pre-specified (primary and secondary) outcomes that are of interest in the review have been reported in the pre-specified way
Other bias	Unclear risk	Lack of sufficient information to determine the risk of bias

wang2014

Methods	
Participants	
Interventions	
Outcomes	
Notes	

Risk of bias table

Bias	Authors' judgement	Support for judgement
Random sequence generation (selection bias)	Low risk	using a persudo-number generator
Allocation concealment (selection bias)	Low risk	Using identical placbo tablets.
Blinding of participants and personnel (performance bias)	Low risk	Throughout the study, the persons who administered the medications, the raters, and the patients were all blind to medication assignments.
Blinding of outcome assessment (detection bias)	Low risk	Outcomes are unlikely to be affected by blindness
Incomplete outcome data (attrition bias)	Low risk	No missing data for the outcome
Selective reporting (reporting bias)	Low risk	The study protocol is available and all of the study ˀ s pre-specified (primary and secondary) outcomes that are of interest in the review have been reported in the pre-specified way
Other bias	Low risk	There is no obvious other bias.

wong2003

Methods	
Participants	
Interventions	
Outcomes	
Notes	

Risk of bias table

Bias	Authors' judgement	Support for judgement
Random sequence generation (selection bias)	Unclear risk	Insufficient reporting of attrition/exclusions to permit judgement of 'Low risk' or 'High risk'
Allocation concealment (selection bias)	Unclear risk	Insufficient information to permit judgement of 'Low risk' or 'High risk'.
Blinding of participants and personnel (performance bias)	Unclear risk	Insufficient information to permit judgement of 'Low risk' or 'High risk'.
Blinding of outcome assessment (detection bias)	Unclear risk	Insufficient information to permit judgement of 'Low risk' or 'High risk'.
Incomplete outcome data (attrition bias)	Low risk	No missing data for the outcome
Selective reporting (reporting bias)	Low risk	The study protocol is available and all of the study's pre-specified (primary and secondary) outcomes that are of interest in the review have been reported in the pre-specified way
Other bias	Unclear risk	Lack of sufficient information to determine the risk of bias

Zesiewicz2017

Methods	
Participants	
Interventions	
Outcomes	
Notes	

Risk of bias table

Bias	Authors' judgement	Support for judgement
Random sequence generation (selection bias)	Low risk	Insufficient reporting of attrition/exclusions to permit judgement of 'Low risk' or 'High risk'

Allocation concealment (selection bias)	Low risk	Ropinirole PR and placebo tablets were identical in appearance.
Blinding of participants and personnel (performance bias)	Unclear risk	Insufficient information to permit judgement of ‘Low risk’ or ‘High risk’.
Blinding of outcome assessment (detection bias)	Low risk	Outcomes are unlikely to be affected by blindness
Incomplete outcome data (attrition bias)	Low risk	No missing data for the outcome
Selective reporting (reporting bias)	Low risk	The study protocol is available and all of the study’s pre-specified (primary and secondary) outcomes that are of interest in the review have been reported in the pre-specified way
Other bias	Unclear risk	Lack of sufficient information to determine the risk of bias

Zhang2013

Methods	
Participants	
Interventions	
Outcomes	
Notes	

Risk of bias table

Bias	Authors' judgement	Support for judgement
Random sequence generation (selection bias)	Low risk	Participants were randomized in a ratio of 1:1 to rasagiline or placebo groups, according to a computer-generated randomization code that was generated by an independent third party (Department of Statistics of the Third Military Medical University).
Allocation concealment (selection bias)	Low risk	and placebo tablets were identical in appearance, including size, colour, shape and packaging.
Blinding of participants and personnel (performance bias)	Low risk	Patients, investigators and coordinating staffwere blindedto groupassignments.
Blinding of outcome assessment (detection bias)	Unclear risk	not mentioned in the paper.
Incomplete outcome data (attrition bias)	Low risk	unlikely to happen
Selective reporting (reporting bias)	Low risk	unlikely to happen

Other bias	Low risk	unlikely to happen

zhang2017

Methods	
Participants	
Interventions	
Outcomes	
Notes	

Risk of bias table

Bias	Authors' judgement	Support for judgement
Random sequence generation (selection bias)	Low risk	Following screening (Visit-1), patients were randomized 1:1 to rotigotine or placebo using an interactive voice/web response [illegible]
Allocation concealment (selection bias)	Low risk	Active and placebo patches were matched in size and appearance
Blinding of participants and personnel (performance bias)	Low risk	Unlikely to happen.
Blinding of outcome assessment (detection bias)	Low risk	Outcomes are unlikely to be affected by blindness
Incomplete outcome data (attrition bias)	Low risk	No missing data for the outcome
Selective reporting (reporting bias)	Low risk	The study protocol is available and all of the study’s pre-specified (primary and secondary) outcomes that are of interest in the review have been reported in the pre-specified
Other bias	Low risk	There is no obvious other bias.

Footnotes

Table 1 Estimates of effects and quality ratings for comparison of different treatment to ‘On’ time without troublesome dyskinesia

	**Direct evidence**		**Indirect evidence**		**Network meta-analysis**	
**comparison**	**Mean Difference (95% CrI)**	**Quality of evidence**	**Mean Difference (95% CrI)**	**Quality of evidence**	**Mean Difference (95% CrI)**	**Quality of evidence**
Ropinirole_IR vs. Placebo	0.70 (0.076, 1.4)	Moderate*	0.44 (−0.78, 1.6)	Moderate*	0.64 (0.15, 1.1)	Moderate*
Sumanirole vs. Placebo			-0.54 (-1.25, 0.21)	High	-0.54 (-1.25, 0.21)	High
Apomorphine vs. Placebo			1.97 (0.64, 3.31)	Low	1.97 (0.64, 3.31)	Low
Rotigotine vs. Placebo			-0.70 (-1.16, -0.27)	Moderate*	-0.70 (-1.16, -0.27)	Moderate*
Pramipexole vs. Placebo			1.04 (0.18, 1.86)	High	1.04 (0.18, 1.86)	High
Ropinirole_PR vs. Placebo	0.73 (0.22, 1.2)	High	0.98 (−0.20, 2.1)	High	0.79 (0.34, 1.2)	High
Pramipexole vs. Rotigotine			-0.35 (-1.18, 0.55)	High	-0.35 (-1.18, 0.55)	High
Ropinirole_PR vs. Ropinirole_IR	0.30 (−0.70, 1.3)	Moderate*	0.062 (−0.73, 0.76)	Moderate*	0.15 (−0.42, 0.69)	Moderate*
Ropinirole_IR vs. Rotigotine	0.10 (−1.2, 1.4)	Low*‡	0.077 (−0.64, 0.88)	Low*‡	0.075 (−0.52, 0.69)	Low*‡
Ropinirole_IR vs. Sumanirole			0.10 (-0.67, 0.85)	High	0.10 (-0.67, 0.85)	High

*Limitations (risk of bias). ‡Imprecision.

Table 2 Estimates of effects and quality ratings for comparison of different treatment to ‘off’ time

	Direct evidence		Indirect evidence		Network meta-analysis	
**comparison**	**Mean Difference (95% Crl)**	**Quality of evidence**	**Mean Difference (95% Crl)**	**Quality of evidence**	**Mean Difference (95% Crl)**	**Quality of evidence**
Placebo vs. Ropinirole_IR	−0.71 (−1.4, −0.11)	Moderate*	−1.2 (−2.8, 0.35)	Moderate*	−0.74 (−1.3, −0.29)	Moderate*
Placebo vs. Sumanirole			0.47(-0.17,1.15)	High	0.47(-0.17,1.15)	High
Placebo vs. Ropinirole_PR	−1.6 (−2.1, −0.96)	High	−1.0 (−2.5, 0.51)	High	−1.5 (−2.0, −0.94)	High
Placebo vs. Apomorphine			1.88(0.48, 3.26)	Low*‡	1.88(0.48, 3.26)	Low
Plaebo vs. Pramipexole_ER	−0.70 (−1.4, −0.019)	High	−1.5 (−2.4, −0.48)	High	−0.93 (−1.6, −0.34)	High
Plaebo vs. Pramipexole_IR			1.45(0.95,1.98)	High	1.45(0.95,1.98)	High
Placebo vs. Rotigotine			1.3(0.86,1.72)	Moderate*	1.3(0.86,1.72)	Moderate*
Pramipexole_ER vs.Pramipexole_IR			0.52(0.01,1.01)	High	0.52(0.01,1.01)	High
Pramipexole_IR vs. Rotigotine	0.40 (−0.46, 1.2)	High	−0.11 (−0.87, 0.62)	High	0.15 (−0.45, 0.81)	High
Ropinirole_IR vs. Ropinirole_PR	−0.29 (−1.7, 1.1)	Moderate*	−0.90 (−1.6, −0.069)	Moderate*	−0.77 (−1.4, −0.033)	Moderate*
Ropinirole_IR vs. Rotigotine	−0.50 (−1.4, 0.45)	Moderate*	−0.60 (−1.5, 0.46)	Moderate*	−0.56 (−1.1, 0.068)	Moderate*
Sumanirole vs. Ropinirole_IR			-0.26(-0.95,0.36)	High	-0.26(-0.95,0.36)	High

*Limitations (risk of bias). ‡Imprecision.

Appendix 4. Network Meta-analysis for Secondary Outcomes

Table 1 NMA results for the secondary outcomes ‘On’ time (upper triangle) and ‘off’ time (lower triangle) (A), UPDRS III(upper triangle) and UPDRS II(lower triangle)(B), TEAE(lower triangle)(C). MDs < 0 indicated that the treatment specified in the column is more efficacious. Bold underlined results indicate statistical significance.

A	ON							
OFF	Apomorphine							
	**-1.88(-3.26,-0.48)**	Placebo		**1.69(0.81, 2.59)**	**1.13(0.70, 1.80)**	**1.80(0.82, 2.78)**	**1.37(0.74, 2.09)**	0.59(-0.15, 1.47)
	-0.94(-2.44,0.59)	**0.94(0.34,1.56)**	Pramipexole_ER					
	-0.42(-1.9,1.07)	**1.45(0.95,1.98)**	**0.52(0.01,1.01)**	Pramipexole_IR	-0.53(-1.47,0.53)	0.11(-1.22,1.42)	-0.31(-1.21,0.62)	-1.08(-2.22,0.11)
	-1.14(-2.58,0.36)	**0.73(0.29,1.26)**	-0.2(-0.95,0.59)	**-0.72(-1.38,0)**	Ropinirole_IR	0.66(-0.57,1.69)	0.23(-0.58,0.91)	-0.54(-1.41,0.2)
	-0.37(-1.87,1.11)	**1.51(0.94,2.02)**	0.57(-0.29,1.35)	0.06(-0.73,0.76)	**0.78(0.04,1.4)**	Ropinirole_PR	-0.43(-1.59,0.81)	-1.2(-2.41,0.12)
	-0.58(-2.03,0.88)	**1.3(0.86,1.72)**	0.36(-0.38,1.06)	-0.15(-0.8,0.46)	0.56(-0.06,1.1)	-0.21(-0.87,0.49)	Rotigotine	-0.77(-1.76,0.24)
	-1.41(-2.92,0.14)	0.47(-0.17,1.15)	-0.47(-1.36,0.42)	**-0.99(-1.81,-0.15)**	-0.26(-0.95,0.36)	**-1.04(-1.82,-0.16)**	**-0.83(-1.56,-0.04)**	Sumanirole

B	UPDRS II						
UPDRS III	Placebo		**1.47(0.99, 2)**	**1.67(0.94, 2.37)**	**1.67(1, 2.35)**	**2.04(1.43, 2.68)**	**1.44(0.5, 2.38)**
	**4.2(0.7,7.77)**	Pramipexole_ER					
	**5.3(3.95,6.81)**	1.1(-2.4,4.66)	Pramipexole_IR	0.2(-0.68, 1.01)	0.2(-0.65, 1.04)	0.57(-0.15, 1.25)	-0.03(-1.11, 1)
	**4.83(2.99,6.63)**	0.63(-3.38,4.55)	-0.46(-2.85,1.71)	Ropinirole_IR	0.01(-0.88, 0.91)	0.38(-0.45, 1.23)	-0.22(-1.16, 0.72)
	**4.7(2.88,6.63)**	0.50(-3.46,4.51)	-0.6(-2.97,1.7)	-0.14(-2.46,2.35)	Ropinirole_PR	0.37(-0.55, 1.28)	-0.23(-1.38, 0.89)
	**5.25(3.41,7.17)**	1.05(-2.89,5)	-0.05(-2.25,2.05)	0.42(-1.84,2.8)	0.55(-2.09,3.13)	Rotigotine	-0.6(-1.69, 0.47)
	**5.09(1.94,8.21)**	0.88(-3.88,5.52)	-0.21(-3.75,3.13)	0.25(-2.87,3.4)	0.39(-3.27,3.9)	-0.16(-3.78,3.37)	Sumanirole

C								

TEAE	Apomorphine							
	2.36(0.6,4.25)	Placebo						
	2.28(-0.03,4.53)	-0.08(-1.56,1.2)	Pramipexole_ER					
	1.76(-0.38,3.74)	-0.6(-1.74,0.22)	-0.51(-1.98,0.78)	Pramipexole_IR				
	1.92(-0.11,4)	-0.44(-1.42,0.48)	-0.35(-1.97,1.37)	0.15(-1.06,1.65)	Ropinirole_IR			
	1.76(-0.33,3.96)	-0.6(-1.75,0.56)	-0.52(-2.22,1.39)	-0.01(-1.34,1.67)	-0.16(-1.28,1.03)	Ropinirole_PR		
	2.11(-0.08,4.25)	-0.24(-1.49,0.8)	-0.15(-1.97,1.6)	0.36(-1.08,1.89)	0.2(-1.35,1.62)	0.36(-1.38,1.89)	Rotigotine	
	2.12(-0.09,4.39)	-0.25(-1.62,1.08)	-0.16(-2.02,1.85)	0.35(-1.15,2.17)	0.19(-1.14,1.57)	0.35(-1.33,2)	-0.01(-1.67,1.85)	Sumanirole

Table 2 NMA results for the secondary outcome. For NMA results, Log odds ratio lower than 0 indicate that the treatment specified in the column is more tolerability. Underlined results indicate statistical significance

**A.Dykinestic**								
Apomorphine								
26.22(7.7,66.19)	Placebo							
25.28(6.69,65.35)	***-0.92(-1.74,-0.09)***	Pramipexole_ER						
25.13(6.58,65.11)	***-1.1(-1.58,-0.68)***	-0.19(-1.02,0.6)	Pramipexole_IR					
25(6.39,64.91)	***-1.24(-1.92,-0.7)***	-0.32(-1.4,0.61)	-0.13(-0.91,0.53)	Ropinirole_IR				
24.23(5.67,64.2)	***-1.95(-2.81,-1.23)***	-1.04(-2.22,0.07)	***-0.85(-1.78,0)***	-0.72(-1.51,0.09)	Ropinirole_PR			
25.06(6.52,65)	***-1.15(-1.75,-0.65)***	-0.23(-1.23,0.67)	-0.04(-0.69,0.54)	0.09(-0.55,0.79)	0.81(-0.07,1.73)	Rotigotine		

25.07(6.48,65.01)	***-1.18(-2.11,-0.3)***	-0.26(-1.49,0.92)	-0.08(-1.07,0.91)	0.06(-0.74,0.96)	0.78(-0.29,1.91)	-0.03(-1.01,0.99)	Sumanirole	
**B.Fall**								
Placebo								
0.88(-0.37, 2.16)	Ropinirole_PR							
0.10(-0.90, 1.09)	-0.78(-2.42, 0.81)	Rotigotine						
-0.63(-1.91, 0.62)	-1.51(-3.32, 0.24)	-0.73(-2.35, 0.88)	Safinamide					
**C.Gastrointestinal**								
Apomorphine								
1.07(-0.31,2.54)	Placebo							
0.98(-0.55,2.6)	-0.09(-0.76,0.61)	Pramipexole_ER						
0.65(-0.79,2.16)	***-0.42(-0.82,-0.05)***	-0.32(-1.03,0.3)	Pramipexole_IR					
0.58(-0.85,2.13)	***-0.51(-0.95,-0.02)***	-0.41(-1.24,0.41)	-0.08(-0.65,0.55)	Ropinirole_IR				
1.07(-0.39,2.64)	-0.01(-0.53,0.57)	0.09(-0.78,0.97)	0.41(-0.22,1.12)	0.49(-0.06,1.06)	Ropinirole_PR			
0.2(-1.28,1.7)	***-0.88(-1.36,-0.47)***	***-0.77(-1.65,-0.03)***	-0.45(-1.03,0.07)	-0.37(-0.99,0.14)	-0.86(-1.61,-0.23)	Rotigotine		
1.05(-0.57,2.73)	-0.03(-0.85,0.79)	0.07(-1.03,1.11)	0.39(-0.51,1.3)	0.48(-0.48,1.4)	-0.03(-1.03,0.92)	0.85(-0.06,1.81)	Safinamide	
0.32(-1.39,2.14)	-0.76(-1.79,0.34)	-0.67(-1.92,0.6)	-0.34(-1.42,0.83)	-0.26(-1.2,0.71)	-0.74(-1.86,0.35)	0.1(-0.91,1.3)	-0.73(-2.03,0.64)	Sumanirole
**D.Hallucinosis**								
Placebo								
-1.66(-4.22,0.46)	Pramipexole_ER							
-1.31(-2.74,-0.19)	0.35(-1.83,2.64)	Pramipexole_IR						
-1.21(-3.63,1.23)	0.44(-2.67,4)	0.09(-2.48,2.96)	Ropinirole_IR					
-2.51(-5.97,0.9)	-0.85(-4.8,3.48)	-1.2(-4.72,2.58)	-1.29(-3.81,1.13)	Ropinirole_PR				

-1.28(-3.23,0.3)	0.39(-2.36,3.18)	0.04(-1.85,1.82)	-0.05(-3.26,2.73)	1.23(-2.8,4.93)	Rotigotine			
-1.21(-3.65,1.2)	0.43(-2.71,4.03)	0.09(-2.48,2.98)	-0.01(-2.33,2.33)	1.29(-2.02,4.73)	0.05(-2.73,3.25)	Sumanirole		
**E.OtherAE**								
Apomorphine								
2.07(0.82,3.69)	Placebo							
1.89(0.49,3.61)	-0.18(-0.8,0.43)	Pramipexole_ER						
1.71(0.43,3.35)	***-0.36(-0.65,-0.07)***	-0.18(-0.74,0.39)	Pramipexole_IR					
1.6(0.31,3.25)	***-0.48(-0.81,-0.13)***	-0.29(-0.99,0.41)	-0.12(-0.56,0.33)	Ropinirole_IR				
1.58(0.28,3.23)	***-0.49(-0.87,-0.11)***	-0.31(-1.02,0.41)	-0.13(-0.6,0.35)	-0.02(-0.39,0.36)	Ropinirole_PR			
1.84(0.54,3.49)	-0.23(-0.57,0.09)	-0.04(-0.72,0.61)	0.13(-0.26,0.5)	0.25(-0.24,0.69)	0.26(-0.23,0.75)	Rotigotine		
1.39(0.02,3.07)	***-0.68(-1.23,-0.16)***	-0.51(-1.32,0.31)	-0.32(-0.94,0.28)	-0.21(-0.74,0.3)	-0.19(-0.81,0.41)	-0.46(-1.06,0.18)	Sumanirole	

Appendix 5. Evaluation of Inconsistency and Node-Split Analysis of Category-based Network Meta-analysis

Table 1. Evaluation of Inconsistency of primary outcomes

	**consistency model**			**inconsistency model**		
	**Model fit (residual deviance)**			**Model fit (residual deviance)**		
	**Dbar**	**pD**	**DIC**	**Dbar**	**pD**	**DIC**
**'ON' time without troublesome dyskinesia**	35.05228	26.18672	61.239	35.05542	27.53159	62.58701
	35 data points, ratio 1.001, I^2 = 3%			35 data points, ratio 1.002, I^2 = 3%		
**'OFF' time**	31.03824	26.05309	57.09133	31.60075	27.90336	59.50411
	32 data points, ratio 0.9699, I^2 = 0.1%			32 data points, ratio 0.9875, I^2 = 2%		
**'ON' time**	13.40586	12.53115	25.93701	13.4107	12.51853	25.92924
	15 data points, ratio 0.8937, I^2 = 0%			15 data points, ratio 0.894, I^2 = 0%		
**UPDRS III**	33.12997	28.42821	61.55818	32.90998	28.91835	61.82833
	34 data points, ratio 0.9744, I^2 = 0.4%			34 data points, ratio 0.9679, I^2 = 0%		
**UPDRS II**	21.36042	17.98648	39.3469	21.87332	19.03207	40.90539
	24 data points, ratio 0.89, I^2 = 0%			24 data points, ratio 0.9114, I^2 = 0%		

Table 2. Node split analysis of category-based network meta-analysis

'ON' time without troublesome dyskinesia

Study	P−value	Mean Difference (95% CrI)
**Pramipexole_ER vs Placebo**		
direct		−0.70 (−1.4, −0.019)
indirect	0.17825	−1.5 (−2.4, −0.48)
network		−0.93 (−1.6, −0.34)
**Ropinirole_IR vs Placebo**		
direct		−0.71 (−1.4, −0.11)
indirect	0.502975	−1.2 (−2.8, 0.35)
network		−0.74 (−1.3, −0.29)
**Ropinirole_PR vs Placebo**		
direct		−1.6 (−2.1, −0.96)
indirect	0.466675	−1.0 (−2.5, 0.51)
network		−1.5 (−2.0, −0.94)
**Rotigotine vs Pramipexole_IR**		
direct		0.40 (−0.46, 1.2)
indirect	0.346475	−0.11 (−0.87, 0.62)
network		0.15 (−0.45, 0.81)
**Ropinirole_PR vs Ropinirole_IR**		
direct		−0.29 (−1.7, 1.1)
indirect	0.4364	−0.90 (−1.6, −0.069)
network		−0.77 (−1.4, −0.033)
**Rotigotine vs Ropinirole_IR**		
direct		−0.50 (−1.4, 0.45)
indirect	0.8696	−0.60 (−1.5, 0.46)
network		−0.56 (−1.1, 0.068)

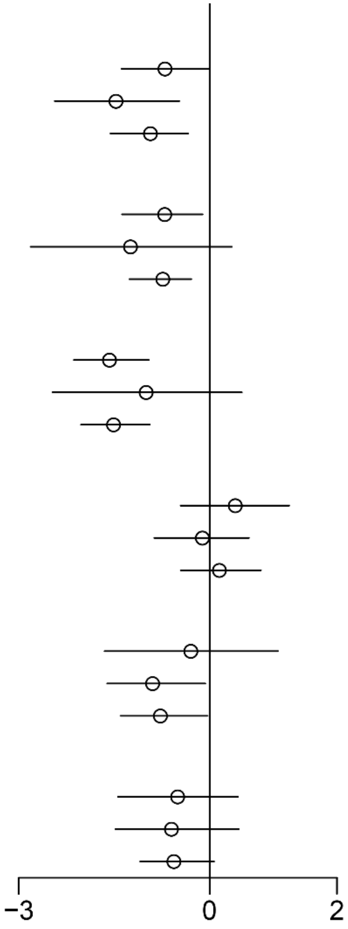

‘OFF’ time

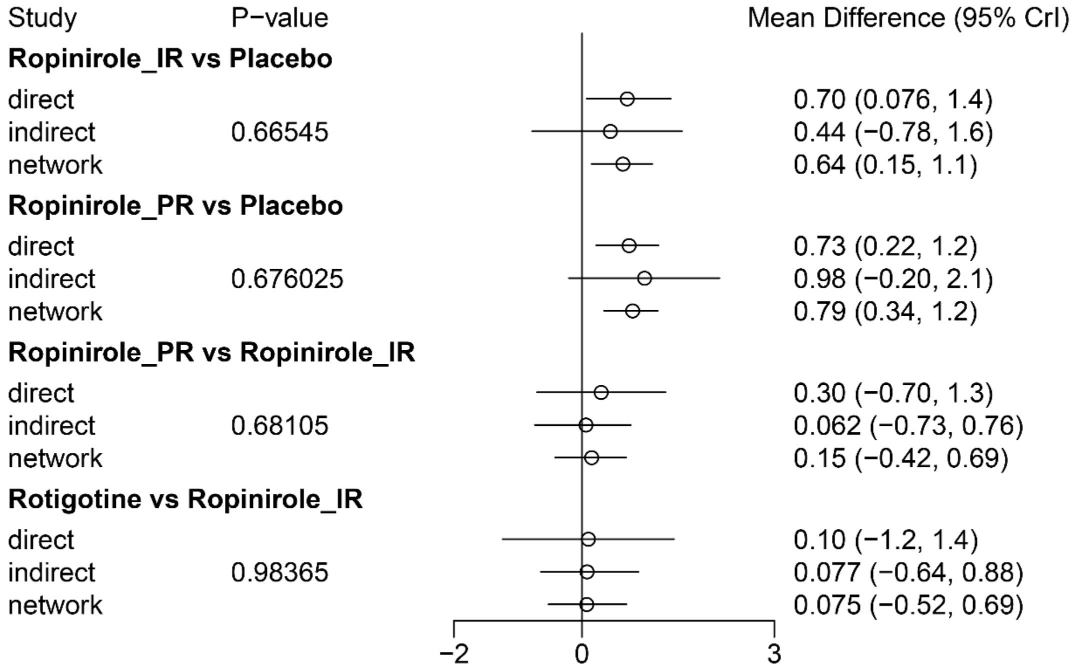

‘ON’ time

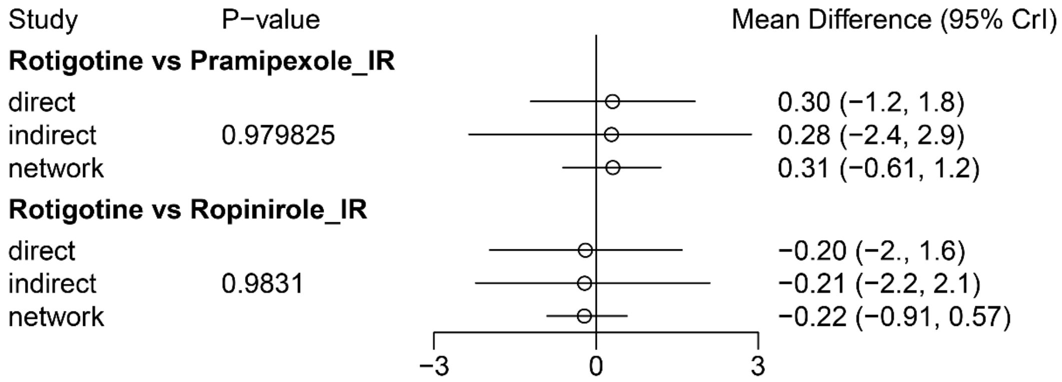

UPDRS III

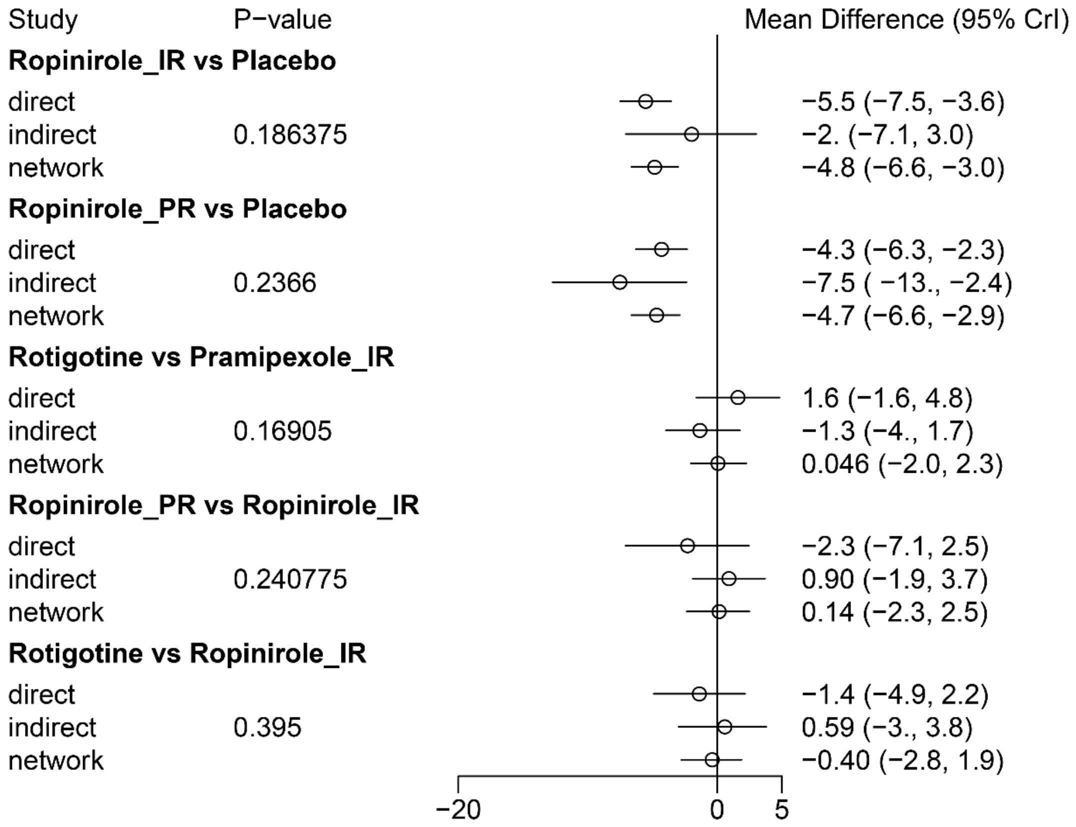
Study
P−value
Mean Difference (95% CrI)
Ropinirole_IR vs Placebo
direct
−5.5 (−7.5, −3.6)
indirect
0.186375
−2. (−7.1, 3.0)
network
−4.8 (−6.6, −3.0)
Ropinirole_PR vs Placebo
direct
−4.3 (−6.3, −2.3)
indirect
0.2366
−7.5 (−13., −2.4)
network
−4.7 (−6.6, −2.9)
Rotigotine vs Pramipexole_IR
direct
1.6 (−1.6, 4.8)
indirect
0.16905
−1.3 (−4., 1.7)
network
0.046 (−2.0, 2.3)
Ropinirole_PR vs Ropinirole_IR
direct
−2.3 (−7.1, 2.5)
indirect
0.240775
0.90 (−1.9, 3.7)
network
0.14 (−2.3, 2.5)
Rotigotine vs Ropinirole_IR
direct
−1.4 (−4.9, 2.2)
indirect
0.395
0.59 (−3., 3.8)
network
−0.40 (−2.8, 1.9)
−20
0
5

UPDRS II

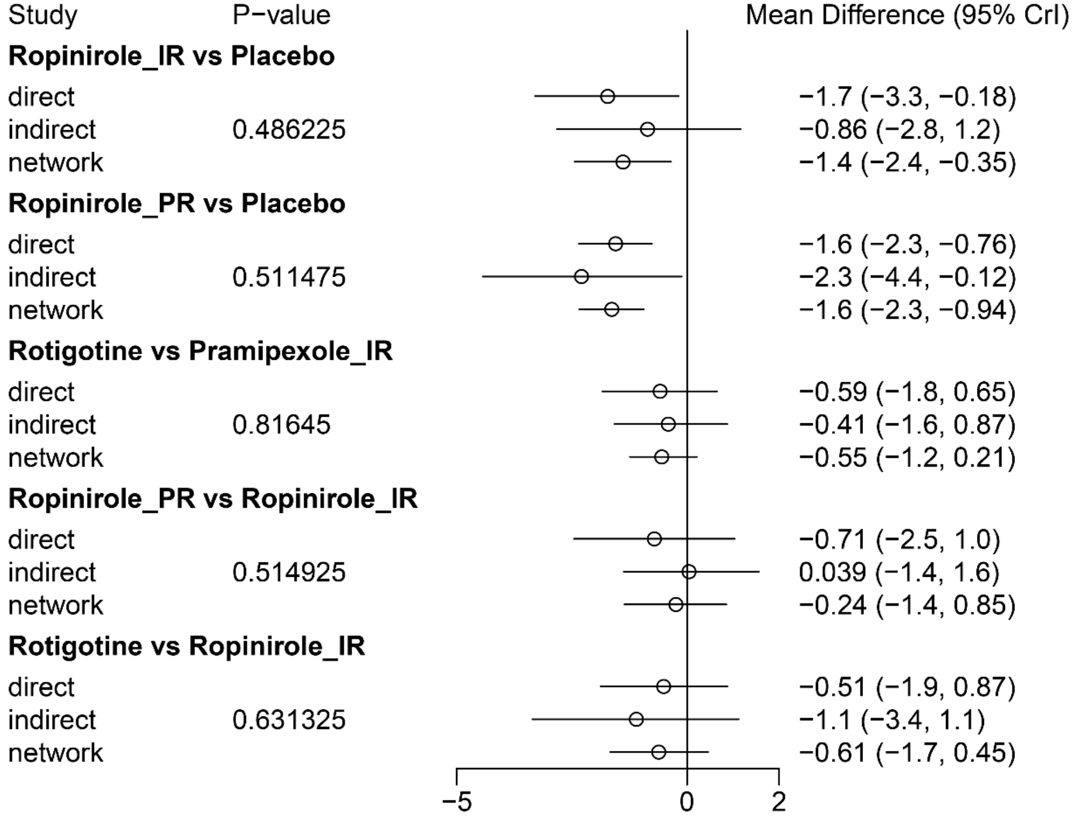
Study
P−value
Mean Difference (95% CrI)
Ropinirole_IR vs Placebo
direct −1.7 (−3.3, −0.18)
indirect 0.486225 −0.86 (−2.8, 1.2)
network −1.4 (−2.4, −0.35)
Ropinirole_PR vs Placebo
direct −1.6 (−2.3, −0.76)
indirect 0.511475 −2.3 (−4.4, −0.12)
network −1.6 (−2.3, −0.94)
Rotigotine vs Pramipexole_IR
direct −0.59 (−1.8, 0.65)
indirect 0.81645 −0.41 (−1.6, 0.87)
network −0.55 (−1.2, 0.21)
Ropinirole_PR vs Ropinirole_IR
direct −0.71 (−2.5, 1.0)
indirect 0.514925 0.039 (−1.4, 1.6)
network −0.24 (−1.4, 0.85)
Rotigotine vs Ropinirole_IR
direct −0.51 (−1.9, 0.87)
indirect 0.631325 −1.1 (−3.4, 1.1)
network −0.61 (−1.7, 0.45)
−5
0
2

Appendix 6. Investigation of Small-Study Effects

Figure 1. Small-study effects assessed via comparison-adjusted network funnel plots for 'ON' time without troublesome dyskinesia

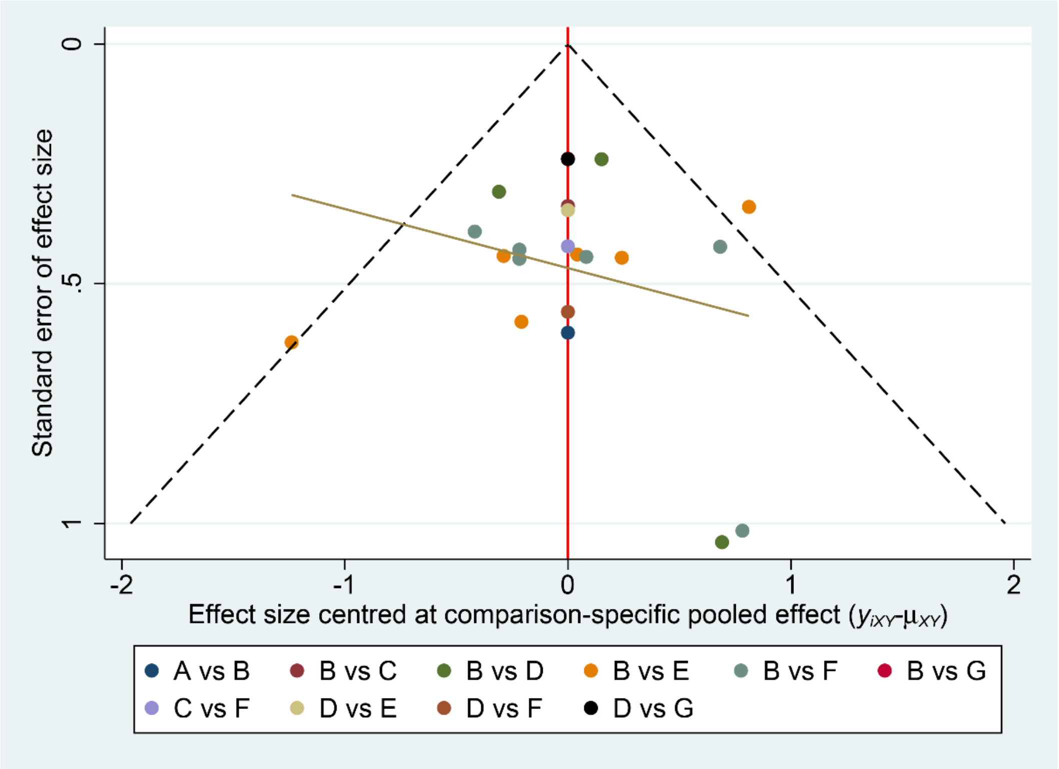

[A Apomorphine, B Ropinirole_PR, C Pramipexole_IR, D Placebo, E Ropinirole_PR, F Rotigotine, G Ropinirole_IR]

In this presentation, all studies are centered on the summary effect estimate of their respective comparisons [**μ**XY (logOR for present study)] which is represented by the vertical red line. Individual study-level effect size is represented by yiXY [where X and Y are two study agents]. Outer dotted lines indicate the triangular region within which 95% of studies are expected to lie in the absence of both biases and heterogeneity (logOR ± 1.96*standard error). Please note that this is drawn only for comparisons with 2 or more studies.

Figure 2. Small-study effects assessed via comparison-adjusted network funnel plots for 'OFF' time

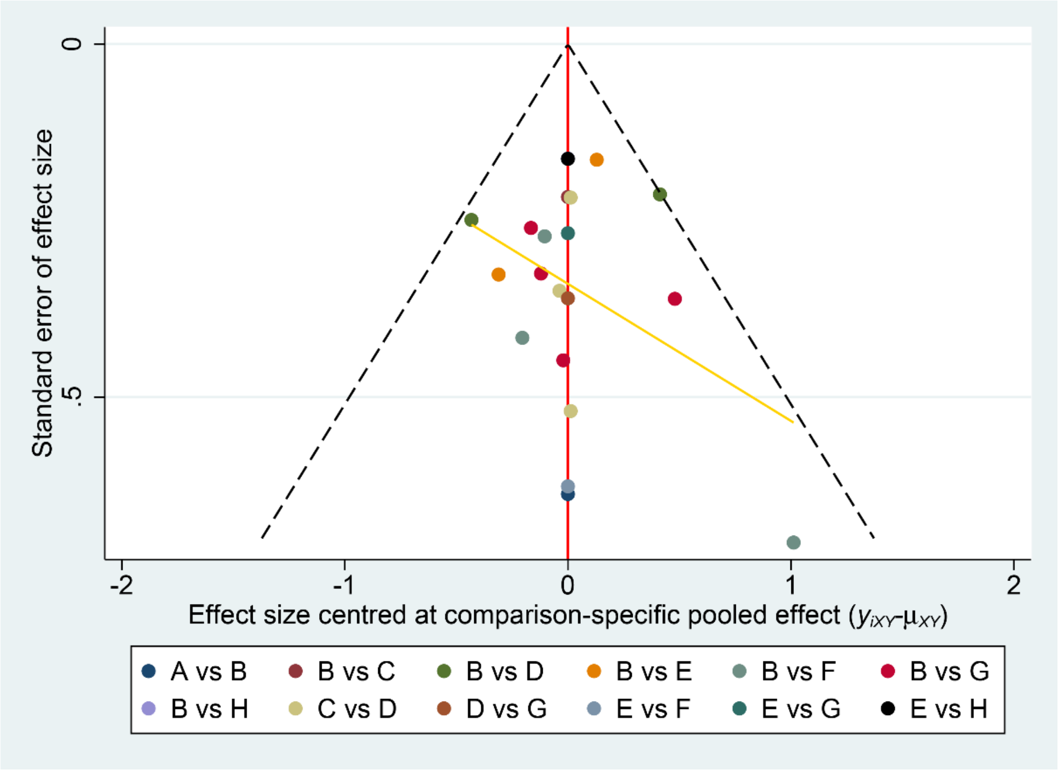

[A: Apomorphine, B: Cabergoline, C: Placebo, D: Pramipexole_ER, E: Pramipexole_IR, F: Ropinirole_IR, G: Ropinirole_PR, H: Rotigotine, I: Sumanirole]

In this presentation, all studies are centered on the summary effect estimate of their respective comparisons [**μ**XY (logOR for present study)] which is represented by the vertical red line. Individual study-level effect size is represented by yiXY [where X and Y are two study agents]. Outer dotted lines indicate the triangular region within which 95% of studies are expected to lie in the absence of both biases and heterogeneity (logOR ± 1.96*standard error). Please note that this is drawn only for comparisons with 2 or more studies.

Figure 3. Small-study effects assessed via comparison-adjusted network funnel plots for 'ON' time

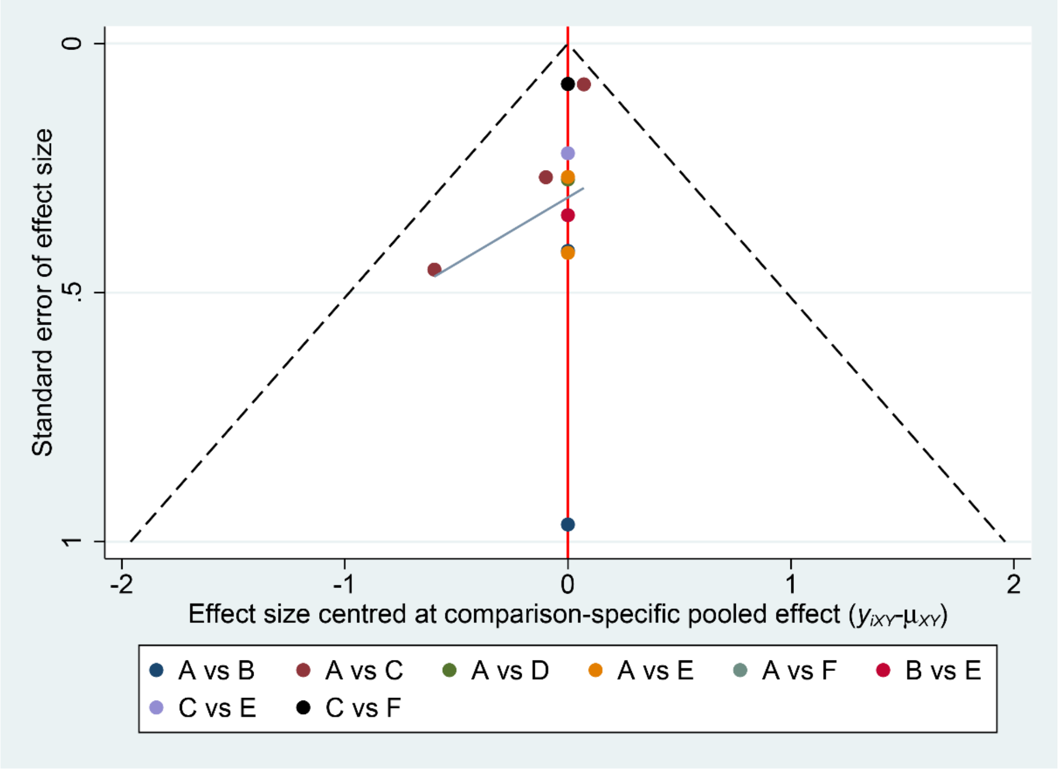

[A Placebo, B: Pramipexole_IR, C:Ropinirole_IR, D: Ropinirole_PR, E: Rotigotine, F: Sumanirole]

In this presentation, all studies are centered on the summary effect estimate of their respective comparisons [**μ**XY (logOR for present study)] which is represented by the vertical red line. Individual study-level effect size is represented by yiXY [where X and Y are two study agents]. Outer dotted lines indicate the triangular region within which 95% of studies are expected to lie in the absence of both biases and heterogeneity (logOR ± 1.96*standard error). Please note that this is drawn only for comparisons with 2 or more studies.

Figure 4. Small-study effects assessed via comparison-adjusted network funnel plots for UPDRS III

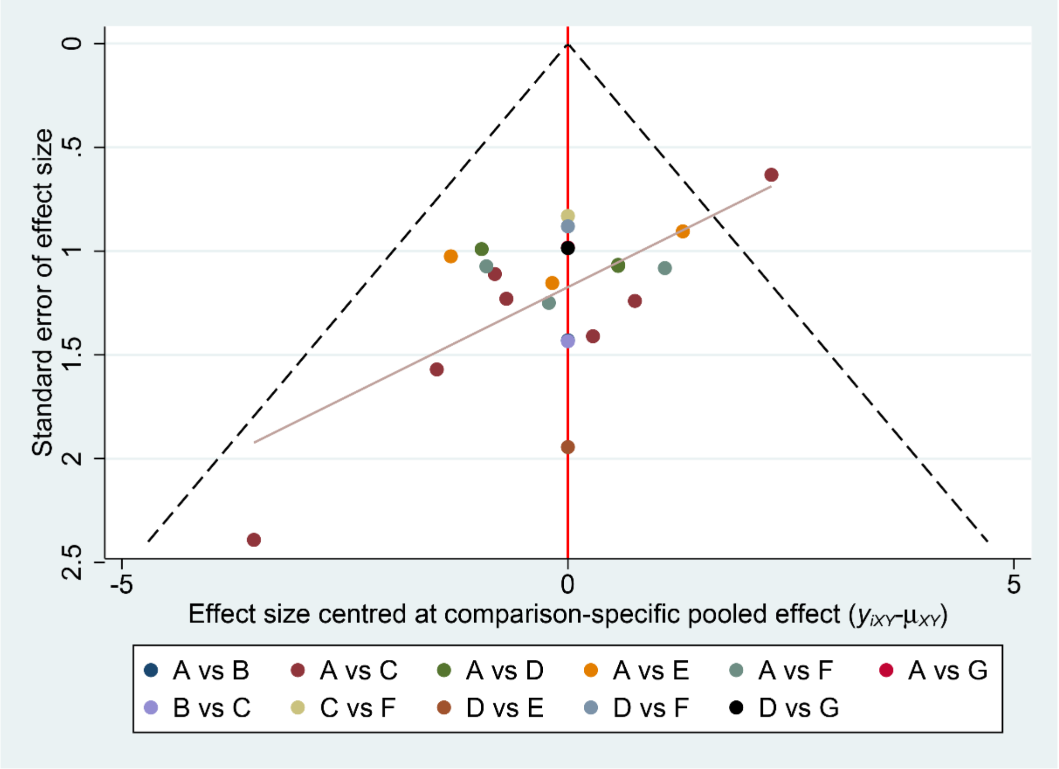

[A Placebo, B: Pramipexole_ER, C: Pramipexole_IR, D: Ropinirole_IR, E: Ropinirole_PR, F: Rotigotine, G: Sumanirole]

In this presentation, all studies are centered on the summary effect estimate of their respective comparisons [**µ**XY (logOR for present study)] which is represented by the vertical red line. Individual study-level effect size is represented by yiXY [where X and Y are two study agents]. Outer dotted lines indicate the triangular region within which 95% of studies are expected to lie in the absence of both biases and heterogeneity (logOR ± 1.96*standard error). Please note that this is drawn only for comparisons with 2 or more studies.

Figure 5. Small-study effects assessed via comparison-adjusted network funnel plots for UPDRS II

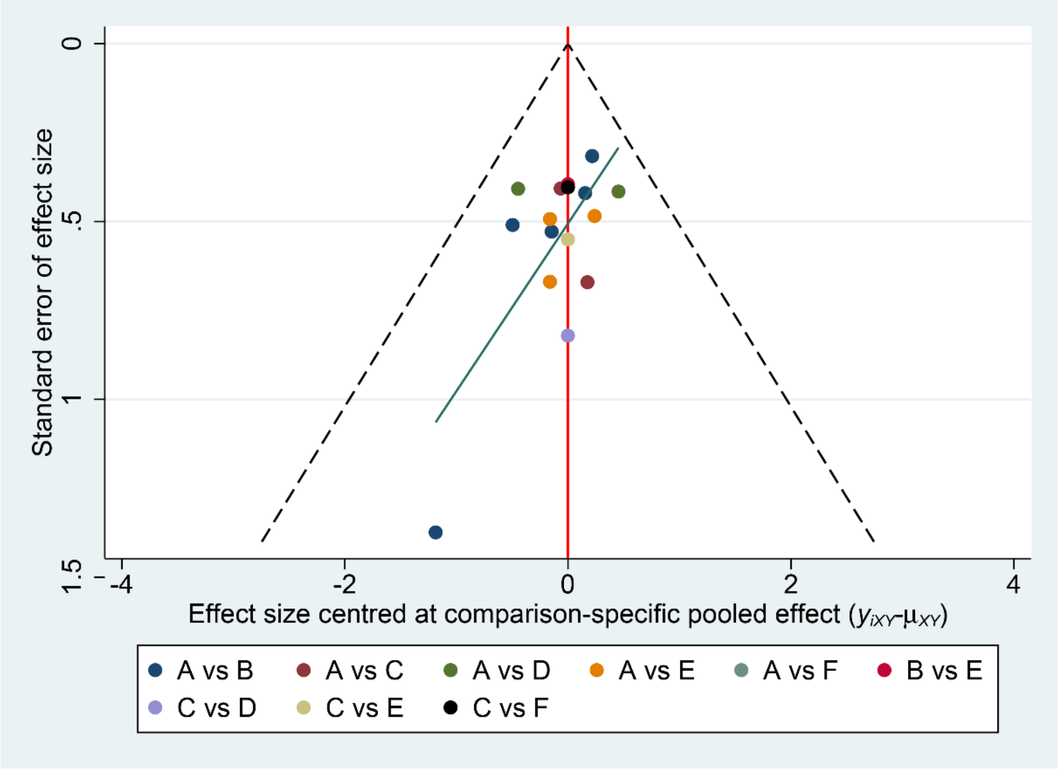

[A: Placebo, B: Pramipexole_IR, C: Ropinirole_IR, D: Ropinirole_PR, E: Rotigotine, F: Sumanirole]

In this presentation, all studies are centered on the summary effect estimate of their respective comparisons [**µ**XY (logOR for present study)] which is represented by the vertical red line. Individual study-level effect size is represented by yiXY [where X and Y are two study agents]. Outer dotted lines indicate the triangular region within which 95% of studies are expected to lie in the absence of both biases and heterogeneity (logOR ± 1.96*standard error). Please note that this is drawn only for comparisons with 2 or more studies.

Appendix 7. Meta-regression Analysis for Outcomes of 'OFF time'

Network meta-regression coefficients based on age

Results on the Mean Difference scale

Iterations = 20001:70000

Thinning interval = 1

Number of chains = 4

Sample size per chain = 50000

1. Empirical mean and standard deviation for each variable,
   plus standard error of the mean:

	Mean	SD	Naive SE	Time-series SE
d.Placebo.Apomorphine	-1.97441	1.7766	0.0039725	0.090160
d.Placebo.Pramipexole_ER	-0.62878	1.3198	0.0029512	0.098586
d.Placebo.Pramipexole_IR	-1.03304	1.3164	0.0029437	0.099887
d.Placebo.Ropinirole_IR	-0.89385	0.1954	0.0004370	0.002243
d.Placebo.Ropinirole_PR	-1.19142	0.3249	0.0007265	0.005053
d.Placebo.Rotigotine	-1.33510	0.3505	0.0007838	0.005871
d.Placebo.Sumanirole	-0.50843	0.3956	0.0008847	0.013979
sd.d	0.19221	0.1278	0.0002858	0.002173
beta[1]	-0.61028	10.0086	0.0223798	0.567000
beta[3]	0.07501	1.6170	0.0036157	0.119793
beta[4]	0.09030	1.6163	0.0036141	0.124848
beta[5]	-0.47224	1.1128	0.0024882	0.014854
beta[6]	0.76887	0.6028	0.0013478	0.008570
beta[7]	0.10188	0.8436	0.0018864	0.013140
beta[8]	-0.04130	7.4819	0.0167300	0.301206

2. Quantiles for each variable:

	2.5%	25%	50%	75%	97.5%
d.Placebo.Apomorphine	-5.68475	-2.5273	-1.877063	-1.23453	0.9810
d.Placebo.Pramipexole_ER	-3.19646	-1.3471	-0.682271	0.05408	2.1678
d.Placebo.Pramipexole_IR	-3.64107	-1.7577	-1.074440	-0.34441	1.7518
d.Placebo.Ropinirole_IR	-1.28978	-1.0039	-0.896331	-0.78309	-0.4955
d.Placebo.Ropinirole_PR	-1.82499	-1.4056	-1.193634	-0.97614	-0.5489
d.Placebo.Rotigotine	-2.02795	-1.5611	-1.334897	-1.10643	-0.6498
d.Placebo.Sumanirole	-1.35256	-0.6643	-0.502704	-0.34096	0.3414
sd.d	0.01201	0.1019	0.171567	0.25778	0.4949
beta[1]	-22.75865	-1.8925	-0.005206	1.85352	16.3547
beta[3]	-3.10052	-0.7942	0.017252	0.87601	3.5159
beta[4]	-3.10829	-0.7842	0.037086	0.90353	3.5223
beta[5]	-2.74511	-1.1601	-0.450403	0.23513	1.6836

```
beta[6]              -0.40579  0.3704   0.765343  1.16531  1.9641
beta[7]              -1.55361 -0.4492   0.097918  0.65048  1.7778
beta[8]             -17.40929 -2.0307  -0.060953  1.79364 16.9338
```

-- Model fit (residual deviance):

```
    Dbar       pD      DIC
35.82601 29.16850 64.99451
```

35 data points, ratio 1.024, I^2 = 5%

-- Regression settings:

Regression on "Age", unrelated coefficients, "Placebo" as control
Input standardized: x' = (Age - 63.92133) / 3.773213
Estimates at the centering value: Age = 63.92133

Network meta-regression coefficients based on duration

> summary(results)

Results on the Mean Difference scale

Iterations = 20001:70000
Thinning interval = 1
Number of chains = 4
Sample size per chain = 50000

1. Empirical mean and standard deviation for each variable,
   plus standard error of the mean:

```
                               Mean      SD  Naive SE Time-series SE
d.Placebo.Apomorphine       -2.3166 4.19302 0.0093759       0.478051
d.Placebo.Cabergoline       -1.4858 5.35026 0.0119635       0.606544
d.Placebo.Pramipexole_ER    -0.9436 0.43703 0.0009772       0.009219
d.Placebo.Pramipexole_IR    -1.5001 0.17784 0.0003977       0.001830
d.Placebo.Ropinirole_IR     -1.1193 0.15490 0.0003464       0.002267
d.Placebo.Ropinirole_PR     -1.5261 0.50056 0.0011193       0.011609
d.Placebo.Rotigotine        -1.3875 0.14560 0.0003256       0.001722
d.Placebo.Sumanirole         0.1976 3.50865 0.0078456       0.492282
sd.d                         0.1459 0.09003 0.0002013       0.001639
beta[1]                      0.5469 5.40988 0.0120969       0.608229
beta[2]                      0.1645 5.14005 0.0114935       0.600734
beta[4]                     -0.7413 1.06941 0.0023913       0.021701
```

```
beta[5]                   -0.9930 0.59970 0.0013410          0.006379
beta[6]                    0.4932 0.35907 0.0008029          0.005727
beta[7]                    0.5559 1.36502 0.0030523          0.031306
beta[8]                   -0.0317 0.82273 0.0018397          0.004692
beta[9]                   -1.2948 6.68604 0.0149504          0.940264
```

2. Quantiles for each variable:

```
                                2.5%      25%      50%      75%   97.5%
d.Placebo.Apomorphine     -13.994324 -3.66880 -2.06528 -0.49808  5.9359
d.Placebo.Cabergoline     -12.608538 -3.57761 -1.56795  0.44914 11.0943
d.Placebo.Pramipexole_ER   -1.820586 -1.22692 -0.93832 -0.65911 -0.0816
d.Placebo.Pramipexole_IR   -1.854307 -1.61354 -1.50026 -1.38527 -1.1467
d.Placebo.Ropinirole_IR    -1.423186 -1.22234 -1.12002 -1.01695 -0.8118
d.Placebo.Ropinirole_PR    -2.490563 -1.85792 -1.54139 -1.20576 -0.4968
d.Placebo.Rotigotine       -1.672156 -1.48433 -1.38844 -1.29202 -1.0995
d.Placebo.Sumanirole       -6.061830 -1.48625 -0.34774  1.32457  8.8795
sd.d                        0.008512  0.07984  0.13582  0.19819  0.3510
beta[1]                   -10.109222 -1.62113  0.15899  2.10521 15.4972
beta[2]                   -11.929648 -1.56407  0.20372  2.06766 10.9339
beta[4]                    -2.929126 -1.41871 -0.70874 -0.03532  1.2863
beta[5]                    -2.178372 -1.38726 -0.99052 -0.59969  0.1904
beta[6]                    -0.177356  0.25312  0.48075  0.71788  1.2402
beta[7]                    -2.043956 -0.32774  0.49507  1.39571  3.4381
beta[8]                    -1.648454 -0.57243 -0.03334  0.50292  1.5947
beta[9]                   -17.836254 -3.43051 -0.25879  1.90466 10.6418
```

-- Model fit (residual deviance):

```
    Dbar       pD      DIC
36.90187 32.23011 69.13197
```

41 data points, ratio 0.9, I^2 = 0%

-- Regression settings:

Regression on "duration", unrelated coefficients, "Placebo" as control
Input standardized: x' = (duration - 7.404118) / 4.950965
Estimates at the centering value: Age = 7.404118

Network meta-regression coefficients based on follow-up

> summary(results)

Results on the Mean Difference scale

Iterations = 20001:70000
Thinning interval = 1
Number of chains = 4
Sample size per chain = 50000

1. Empirical mean and standard deviation for each variable,
plus standard error of the mean:

	Mean	SD	Naive SE	Time-series SE
d.Placebo.Cabergoline	-0.85606	3.5282	0.0078893	0.342093
d.Placebo.Pramipexole_ER	-0.82797	0.7305	0.0016334	0.023655
d.Placebo.Pramipexole_IR	-1.65419	0.2239	0.0005007	0.002844
d.Placebo.Ropinirole_IR	-0.99860	0.2001	0.0004475	0.003529
d.Placebo.Ropinirole_PR	-1.53894	0.2114	0.0004726	0.002475
d.Placebo.Rotigotine	-1.36786	0.1956	0.0004374	0.002399
d.Placebo.Sumanirole	0.48773	3.2676	0.0073065	0.648943
sd.d	0.17480	0.1074	0.0002400	0.001966
beta[1]	1.45884	11.4445	0.0255907	1.299455
beta[3]	-0.18916	2.4077	0.0053838	0.076410
beta[4]	-1.55882	1.0655	0.0023825	0.012616
beta[5]	0.12239	0.2858	0.0006392	0.005700
beta[6]	-0.06151	0.4303	0.0009622	0.003885
beta[7]	-0.40093	1.4410	0.0032222	0.014860
beta[8]	-1.17392	3.8796	0.0086751	0.777138

2. Quantiles for each variable:

	2.5%	25%	50%	75%	97.5%
d.Placebo.Cabergoline	-6.22754	-2.10058	-1.23135	-0.2973	8.2691
d.Placebo.Pramipexole_ER	-2.42412	-1.22799	-0.80184	-0.3942	0.6043
d.Placebo.Pramipexole_IR	-2.09569	-1.79871	-1.65607	-1.5073	-1.2116
d.Placebo.Ropinirole_IR	-1.39109	-1.13089	-0.99979	-0.8676	-0.6054
d.Placebo.Ropinirole_PR	-1.94889	-1.67937	-1.54104	-1.3997	-1.1198
d.Placebo.Rotigotine	-1.74876	-1.49750	-1.37084	-1.2406	-0.9785
d.Placebo.Sumanirole	-5.20001	-1.67720	-0.02081	2.4477	7.4340
sd.d	0.01336	0.09584	0.16138	0.2367	0.4249
beta[1]	-15.74422	-1.55741	0.13354	2.0977	31.9771
beta[3]	-5.56647	-1.39620	-0.09315	1.1262	4.6193
beta[4]	-3.68968	-2.25539	-1.53853	-0.8489	0.4961
beta[5]	-0.43937	-0.06169	0.12107	0.3046	0.6943
beta[6]	-0.90166	-0.35002	-0.06175	0.2259	0.7887

beta[7]	-3.41982	-1.26199	-0.35067	0.5034	2.3883
beta[8]	-9.42225	-3.49638	-0.56824	1.3845	5.5845

-- Model fit (residual deviance):

Dbar	pD	DIC
38.69718	30.71177	69.40895

39 data points, ratio 0.9922, I^2 = 2%

-- Regression settings:

Regression on " **follow-up**", unrelated coefficients, "Placebo" as control
Input standardized: x' = (**follow-up** - 4.84375) / 6.128825
Estimates at the centering value: **follow-up** = 4.84375

Network meta-regression coefficients based on HY

Results on the Mean Difference scale

Iterations = 20001:70000
Thinning interval = 1
Number of chains = 4
Sample size per chain = 50000

1. Empirical mean and standard deviation for each variable,
   plus standard error of the mean:

	Mean	SD	Naive SE	Time-series SE
d.Placebo.Pramipexole_ER	-0.70679	0.4756	0.0010635	0.010665
d.Placebo.Pramipexole_IR	-1.12870	0.4805	0.0010745	0.011126
d.Placebo.Ropinirole_IR	-0.93089	0.1421	0.0003177	0.001160
d.Placebo.Ropinirole_PR	-0.93696	0.5304	0.0011859	0.009198
d.Placebo.Rotigotine	-1.29917	0.1979	0.0004426	0.001948
d.Placebo.Sumanirole	-0.31383	0.7760	0.0017352	0.064054
sd.d	0.17831	0.1176	0.0002629	0.001780
beta[2]	-0.04412	1.5344	0.0034311	0.037829
beta[3]	-0.06914	1.5528	0.0034721	0.038685
beta[4]	-0.75774	0.6157	0.0013767	0.005232
beta[5]	0.38014	0.7218	0.0016141	0.010621
beta[6]	-0.44371	0.3955	0.0008843	0.002416
beta[7]	2.05947	9.0772	0.0202973	0.801491

2. Quantiles for each variable:

	2.5%	25%	50%	75%	97.5%
d.Placebo.Pramipexole_ER	-1.67663	-0.99566	-0.70398	-0.4140	0.2474
d.Placebo.Pramipexole_IR	-2.11613	-1.41482	-1.12543	-0.8328	-0.1643
d.Placebo.Ropinirole_IR	-1.19802	-1.01654	-0.93924	-0.8519	-0.6252
d.Placebo.Ropinirole_PR	-1.98003	-1.29371	-0.93790	-0.5834	0.1006
d.Placebo.Rotigotine	-1.68572	-1.42901	-1.30009	-1.1699	-0.9072
d.Placebo.Sumanirole	-1.39189	-0.65730	-0.46669	-0.2281	2.0720
sd.d	0.01203	0.09432	0.15957	0.2385	0.4645
beta[2]	-3.27385	-0.86949	-0.02284	0.8133	3.0907
beta[3]	-3.31743	-0.90518	-0.04838	0.7934	3.0935
beta[4]	-2.04059	-1.13832	-0.73665	-0.3548	0.4085
beta[5]	-1.01716	-0.09888	0.37037	0.8496	1.8366
beta[6]	-1.21714	-0.70533	-0.44342	-0.1826	0.3378
beta[7]	-10.35537	-1.41632	0.16743	2.2252	30.5811

-- Model fit (residual deviance):

Dbar	pD	DIC
27.18863	24.43769	51.62632

29 data points, ratio 0.9375, I^2 = 0%

-- Regression settings:

Regression on "HY", unrelated coefficients, "Placebo" as control
Input standardized: x' = (HY - 2.62125) / 0.2540624
Estimates at the centering value: **HY** = 2.62125

Network meta-regression coefficients based on male

> summary(results)

Results on the Mean Difference scale

Iterations = 20001:70000
Thinning interval = 1
Number of chains = 4
Sample size per chain = 50000

1. Empirical mean and standard deviation for each variable,
   plus standard error of the mean:

	Mean	SD	Naive SE	Time-series SE
d.Placebo.Apomorphine	-2.19626	3.4712	0.0077619	0.399025
d.Placebo.Pramipexole_ER	-0.77320	0.3187	0.0007126	0.003545
d.Placebo.Pramipexole_IR	-1.24602	0.2321	0.0005189	0.002727
d.Placebo.Ropinirole_IR	-0.96928	0.1312	0.0002933	0.001033
d.Placebo.Ropinirole_PR	-1.86323	0.2977	0.0006657	0.004202
d.Placebo.Rotigotine	-1.38921	0.1523	0.0003405	0.001498
d.Placebo.Sumanirole	-0.50420	2.6843	0.0060022	0.701772
d.Placebo.Zydis_selegiline	-1.39288	1.7952	0.0040143	0.101319
sd.d	0.16528	0.1019	0.0002279	0.001810
beta[1]	1.15172	12.8273	0.0286827	1.613458
beta[3]	-0.53218	0.9967	0.0022287	0.010330
beta[4]	-0.74882	0.4675	0.0010453	0.004599
beta[5]	0.30901	0.2746	0.0006139	0.002552
beta[6]	-2.29394	1.3397	0.0029956	0.018285
beta[7]	0.13457	0.2325	0.0005198	0.001682
beta[8]	0.03947	9.6111	0.0214910	2.659318
beta[9]	-0.64396	5.4478	0.0121817	0.342942

2. Quantiles for each variable:

	2.5%	25%	50%	75%	97.5%
d.Placebo.Apomorphine	-11.6041	-2.70004	-1.90258	-1.13551	3.3186
d.Placebo.Pramipexole_ER	-1.4103	-0.97628	-0.77382	-0.56934	-0.1362
d.Placebo.Pramipexole_IR	-1.7108	-1.39640	-1.24317	-1.09560	-0.7941
d.Placebo.Ropinirole_IR	-1.2212	-1.05055	-0.97300	-0.89170	-0.6959
d.Placebo.Ropinirole_PR	-2.4470	-2.05756	-1.86389	-1.66716	-1.2798
d.Placebo.Rotigotine	-1.6857	-1.48948	-1.39014	-1.29045	-1.0851
d.Placebo.Sumanirole	-6.7049	-1.19150	-0.56259	-0.01295	7.6342
d.Placebo.Zydis_selegiline	-4.6786	-2.21229	-1.54748	-0.84804	3.2854
sd.d	0.0121	0.09157	0.15149	0.22266	0.4035
beta[1]	-18.9458	-1.82529	0.04308	1.98624	36.2941
beta[3]	-2.5482	-1.17672	-0.51045	0.12671	1.3806
beta[4]	-1.6564	-1.05703	-0.75446	-0.44765	0.1889
beta[5]	-0.2280	0.13240	0.30602	0.48421	0.8638
beta[6]	-4.9925	-3.18991	-2.25218	-1.35748	0.1968
beta[7]	-0.3201	-0.01784	0.13427	0.28650	0.5927
beta[8]	-29.1986	-1.64863	0.24144	2.42389	22.2459
beta[9]	-15.1945	-1.89646	-0.13233	1.48409	9.5123

-- Model fit (residual deviance):

Dbar pD DIC

36.89618 31.96750 68.86368

39 data points, ratio 0.9461, I^2 = 0%

-- Regression settings:

Regression on "Age", unrelated coefficients, "Placebo" as control
Input standardized: x' = (Male - 55.71135) / 24.65288
Estimates at the centering value: Male = 55.71135

Network meta-regression coefficients based on UPDRS III
> summary(results)

Results on the Mean Difference scale

Iterations = 20001:70000
Thinning interval = 1
Number of chains = 4
Sample size per chain = 50000

1. Empirical mean and standard deviation for each variable,
   plus standard error of the mean:

	Mean	SD	Naive SE	Time-series SE
d.Placebo.Apomorphine	-3.2439	4.95193	0.0110728	0.743283
d.Placebo.Pramipexole_ER	-0.9649	0.25425	0.0005685	0.002971
d.Placebo.Pramipexole_IR	-1.4707	0.17636	0.0003944	0.001747
d.Placebo.Ropinirole_IR	-1.0807	0.15226	0.0003405	0.001922
d.Placebo.Ropinirole_PR	-1.1503	0.25532	0.0005709	0.003520
d.Placebo.Rotigotine	-1.3423	0.24400	0.0005456	0.004244
d.Placebo.Sumanirole	-1.5185	3.13642	0.0070132	0.687817
sd.d	0.1560	0.09323	0.0002085	0.001618
beta[1]	4.0760	14.90182	0.0333215	2.420942
beta[3]	1.3177	1.46993	0.0032869	0.025375
beta[4]	1.4311	1.37227	0.0030685	0.023149
beta[5]	0.3856	0.31242	0.0006986	0.004845
beta[6]	0.6249	0.28962	0.0006476	0.003149
beta[7]	0.2257	1.65155	0.0036930	0.026189
beta[8]	1.7384	5.22955	0.0116936	1.144435

2. Quantiles for each variable:

	2.5%	25%	50%	75%	97.5%
d.Placebo.Apomorphine	-18.85234	-3.07729	-2.0461	-1.1832	2.5501
d.Placebo.Pramipexole_ER	-1.46655	-1.13263	-0.9644	-0.7966	-0.4673
d.Placebo.Pramipexole_IR	-1.82160	-1.58563	-1.4694	-1.3539	-1.1285
d.Placebo.Ropinirole_IR	-1.37969	-1.17985	-1.0816	-0.9822	-0.7763
d.Placebo.Ropinirole_PR	-1.64820	-1.31981	-1.1512	-0.9805	-0.6433
d.Placebo.Rotigotine	-1.81581	-1.50275	-1.3479	-1.1847	-0.8530
d.Placebo.Sumanirole	-9.74272	-2.55172	-0.8999	0.2805	3.7437
sd.d	0.01130	0.08893	0.1455	0.2099	0.3677
beta[1]	-12.79985	-1.50556	0.3071	2.7151	51.5141
beta[3]	-1.17484	0.30801	1.1513	2.1816	4.6279
beta[4]	-0.91163	0.48164	1.2932	2.2352	4.5017
beta[5]	-0.20350	0.18048	0.3745	0.5795	1.0356
beta[6]	0.05866	0.43122	0.6230	0.8175	1.1982
beta[7]	-3.02369	-0.78614	0.1751	1.2010	3.6916
beta[8]	-7.01489	-1.27318	0.6946	3.4615	15.4816

-- Model fit (residual deviance):

Dbar	pD	DIC
37.33994	31.72856	69.06850

41 data points, ratio 0.9107, I^2 = 0%

-- Regression settings:

Regression on "UPDRS III", unrelated coefficients, "Placebo" as control
Input standardized: x' = (UPDRS III - 27.00059) / 7.005018
Estimates at the centering value: UPDRS III = 27.00059

Appendix 8. Sensitivity Analysis for Outcomes

Table 1. NMA results for sensitivity analyses of the outcome of "OFF time"

Inclusion of all the studies the studies

Apomorphine							
-1.88(-3.26, -0.48)	Placebo						
-0.94(-2.44,0.59)	***0.94(0.34,1.56)***	Pramipexole_ER					
-0.42(-1.9,1.07)	***1.45(0.95,1.98)***	***0.52(0.01,1.01)***	Pramipexole_IR				
-1.14(-2.58,0.36)	***0.73(0.29,1.26)***	-0.2(-0.95,0.59)	***-0.72(-1.38,0)***	Ropinirole_IR			
-0.37(-1.87,1.11)	***1.51(0.94,2.02)***	0.57(-0.29,1.35)	0.06(-0.73,0.76)	***0.78(0.04,1.4)***	Ropinirole_PR		
-0.58(-2.03,0.88)	***1.3(0.86,1.72)***	0.36(-0.38,1.06)	-0.15(-0.8,0.46)	0.56(-0.06,1.1)	-0.21(-0.87,0.49)	Rotigotine	
-1.41(-2.92,0.14)	0.47(-0.17,1.15)	-0.47(-1.36,0.42)	***-0.99(-1.81,-0.15)***	-0.26(-0.95,0.36)	***-1.04(-1.82,-0.16)***	***-0.83(-1.56,-0.04)***	Sumanirole

Exclusion of the studies that were published after 2000

Apomorphine							
-1.89(-3.27,-0.49)	Placebo						
-0.95(-2.47,0.57)	***0.93(0.34,1.56)***	Pramipexole_ER					
-0.43(-1.9,1.05)	***1.45(0.95,1.97)***	***0.52(0.01,1.01)***	Pramipexole_IR				
-1.14(-2.6,0.34)	***0.74(0.28,1.26)***	-0.19(-0.95,0.6)	-0.71(-1.39,0.01)	Ropinirole_IR			
-0.37(-1.89,1.1)	***1.51(0.94,2.03)***	0.58(-0.29,1.35)	0.06(-0.73,0.77)	***0.77(0.03,1.4)***	Ropinirole_PR		
-0.59(-2.05,0.85)	***1.29(0.85,1.72)***	0.36(-0.39,1.06)	-0.16(-0.81,0.46)	0.56(-0.07,1.09)	-0.22(-0.88,0.48)	Rotigotine	
-1.41(-2.94,0.13)	0.47(-0.17,1.15)	-0.46(-1.36,0.44)	***-0.99(-1.81,-0.14)***	-0.27(-0.95,0.37)	***-1.05(-1.82,-0.15)***	***-0.83(-1.56,-0.04)***	Sumanirole

Exclusion of studies that had reporting bias

Apomorphine							
-1.88(-3.26,-0.48)	Placebo						
-0.94(-2.44,0.59)	***0.94(0.34,1.56)***	Pramipexole_ER					
-0.42(-1.9,1.07)	***1.45(0.95,1.98)***	***0.52(0.01,1.01)***	Pramipexole_IR				
-1.14(-2.58,0.36)	***0.73(0.29,1.26)***	-0.2(-0.95,0.59)	***-0.72(-1.38,0)***	Ropinirole_IR			
-0.37(-1.87,1.11)	***1.51(0.94,2.02)***	0.57(-0.29,1.35)	0.06(-0.73,0.76)	***0.78(0.04,1.4)***	Ropinirole_PR		
-0.58(-2.03,0.88)	***1.3(0.86,1.72)***	0.36(-0.38,1.06)	-0.15(-0.8,0.46)	0.56(-0.06,1.1)	-0.21(-0.87,0.49)	Rotigotine	
-1.41(-2.92,0.14)	0.47(-0.17,1.15)	-0.47(-1.36,0.42)	***-0.99(-1.81,-0.15)***	-0.26(-0.95,0.36)	***-1.04(-1.82,-0.16)***	***-0.83(-1.56,-0.04)***	Sumanirole

Exclusion of studies that had attrition bias

Apomorphine							
-1.88(-3.26,-0.48)	Placebo						
-0.94(-2.44,0.59)	***0.94(0.34,1.56)***	Pramipexole_ER					
-0.42(-1.9,1.07)	***1.45(0.95,1.98)***	***0.52(0.01,1.01)***	Pramipexole_IR				
-1.14(-2.58,0.36)	***0.73(0.29,1.26)***	-0.2(-0.95,0.59)	***-0.72(-1.38,0)***	Ropinirole_IR			
-0.37(-1.87,1.11)	***1.51(0.94,2.02)***	0.57(-0.29,1.35)	0.06(-0.73,0.76)	***0.78(0.04,1.4)***	Ropinirole_PR		
-0.58(-2.03,0.88)	***1.3(0.86,1.72)***	0.36(-0.38,1.06)	-0.15(-0.8,0.46)	0.56(-0.06,1.1)	-0.21(-0.87,0.49)	Rotigotine	
-1.41(-2.92,0.14)	0.47(-0.17,1.15)	-0.47(-1.36,0.42)	***-0.99(-1.81,-0.15)***	-0.26(-0.95,0.36)	***-1.04(-1.82,-0.16)***	***-0.83(-1.56,-0.04)***	Sumanirole

Exclusion of studies that had selection bias

Placebo						
0.93(0.14,1.74)	Pramipexole_ER					

1.45(0.77,2.12)	0.51(-0.14,1.14)	Pramipexole_IR				
0.67(-0.1,1.56)	-0.26(-1.37,0.94)	-0.77(-1.79,0.36)	Ropinirole_IR			
1.48(0.76,2.09)	0.55(-0.58,1.52)	0.03(-0.97,0.92)	0.8(-0.25,1.63)	Ropinirole_PR		
1.21(0.42,1.92)	0.28(-0.8,1.25)	-0.24(-1.17,0.62)	0.54(-0.69,1.55)	-0.27(-1.25,0.74)	Rotigotine	
0.44(-0.47,1.41)	-0.49(-1.71,0.76)	-1.01(-2.13,0.18)	-0.24(-1.21,0.68)	-1.04(-2.04,0.15)	-0.78(-1.9,0.51)	Sumanirole

Inclusion of studies that lasted 16 weeks or more						
Placebo						
0.92(0.26, 1.63)	Pramipexole_ER					
1.47(0.93, 2.05)	0.55(-0.05, 1.15)	Pramipexole_IR				
0.77(0.28, 1.34)	-0.16(-1, 0.73)	-0.7(-1.44, 0.08)	Ropinirole_IR			
1.51(0.9, 2.05)	0.58(-0.37, 1.42)	0.04(-0.82, 0.79)	0.73(-0.06, 1.4)	Ropinirole_PR		
1.4(0.87, 1.91)	0.47(-0.38, 1.28)	-0.07(-0.81, 0.61)	0.63(-0.07, 1.23)	-0.11(-0.84, 0.68)	Rotigotine	
0.48(-0.22, 1.23)	-0.44(-1.44, 0.56)	-0.99(-1.91, -0.07)	-0.28(-1.03, 0.42)	-1.03(-1.86, -0.06)	-0.92(-1.74, -0.03)	Sumanirole
